# Laser capture microdissection enables transcriptomic analysis of dividing and quiescent liver stages of Plasmodium relapsing species

**DOI:** 10.1111/cmi.12735

**Published:** 2017-03-13

**Authors:** Roger Cubi, Shruthi S. Vembar, Anne Biton, Jean‐Francois Franetich, Mallaury Bordessoulles, Daniel Sossau, Gigliola Zanghi, Henriette Bosson‐Vanga, Magalie Benard, Alicia Moreno, Nathalie Dereuddre‐Bosquet, Roger Le Grand, Artur Scherf, Dominique Mazier

**Affiliations:** ^1^ Centre d'Immunologie et des Maladies Infectieuses, CNRS ERL8255, INSERM U1135 Sorbonne Universités, UPMC Univ Paris 06 Paris France; ^2^ Unité Biologie des Interactions Hôte‐Parasite—Institut Pasteur Paris France; ^3^ CNRS ERL 9195 Paris France; ^4^ INSERM U1201 Paris France; ^5^ Centre de Bioinformatique Biostatistique et Biologie Intégrative (C3BI, USR 3756 Institut Pasteur et CNRS) Paris France; ^6^ Department of Dermatology Eberhard Karls University Tübingen Germany; ^7^ Primacen Normandie Université Mont‐Saint‐Aignan France; ^8^ AP‐HP, Hôpital St. Antoine Service de Parasitologie‐Mycologie 75012 Paris France; ^9^ Immunology of Viral Infections and Autoimmune Diseases CEA—Université Paris Sud 1—INSERM U1184 Fontenay‐aux‐Roses France; ^10^ AP‐HP, Groupe Hospitalier Pitié‐Salpêtrière, Service Parasitologie‐Mycologie Paris France

**Keywords:** hypnozoite, liver stage, Plasmodium, quiescence

## Abstract

Dormant liver stage forms (hypnozoites) of the malaria parasite *Plasmodium vivax* present major hurdles to control and eradicate infection. Despite major research efforts, the molecular composition of hypnozoites remains ill defined. Here, we applied a combination of state‐of‐the‐art technologies to generate the first transcriptome of hypnozoites. We developed a robust laser dissection microscopy protocol to isolate individual *Plasmodium cynomolgi* hypnozoites and schizonts from infected monkey hepatocytes and optimized RNA‐seq analysis to obtain the first transcriptomes of these stages. Comparative transcriptomic analysis identified 120 transcripts as being differentially expressed in the hypnozoite stage relative to the dividing liver schizont, with 69 and 51 mRNAs being up‐ or down‐regulated, respectively, in the hypnozoites. This lead to the identification of potential markers of commitment to and maintenance of the dormant state of the hypnozoite including three transcriptional regulators of the ApiAP2 family, one of which is unique to *P. cynomolgi* and *P. vivax*, and the global translational repressor, eIF2a kinase eIK2, all of which are upregulated in the hypnozoite. Together, this work not only provides a primary experimentally‐derived list of molecular markers of hypnozoites but also identifies transcriptional and posttranscriptional regulation of gene expression as potentially being key to establishing and maintaining quiescence.

## 1 INTRODUCTION

Human malaria, which remains a major threat to global health, is caused by five species of the protozoan Apicomplexan parasite *Plasmodium: P. falciparum, P. vivax, P. knowlesi, P. malariae*, and *P. ovale*. Of these, *P. vivax* and *P.ovale* are capable of producing malaria relapses, consisting of the reappearance of parasites in the blood of individuals who have not been exposed to seasonal infectious mosquito bites, weeks, months or even years after the last infection. This is attributed to the development of a dormant stage called hypnozoite in the liver of infected individuals, which is different from the dividing liver schizont that produces up to 10,000 daughter cells from a single mosquito‐derived sporozoite. Since the first description of hypnozoite forms in 1980, *P. vivax* and *P. ovale* hypnozoite research has been limited to in vitro studies in primary hepatocytes, hepatoma cell lines and more recently, in humanized mouse models (Galinski, Meyer, & Barnwell, 2013; Mazier et al., 1984). These studies have been complemented by work on *P. cynomolgi*, which readily infects rhesus monkeys *Macaca mulatta* and *M. fascicularis* and being the closest relative of *P. vivax* (Waters, Higgins, & McCutchan, 1993), is used to model human *P. vivax* infection. Based on decades of research, the following features have become evident: The hypnozoite is a small and round uninucleate form around 5 μm in diameter that is present in the cytoplasm of hepatocytes. It does not undergo DNA replication and can be identified by immunostaining with anti‐Hsp70 antibodies. Its size increases slowly over time in cultured hepatocytes but always remains smaller than the dividing schizont. However, molecular markers that are specific to the hypnozoite remain unknown, as does the molecular basis of hypnozoite commitment and activation.

Our laboratory, using *M. mulatta* and *M. fascicularis* primary hepatocytes*,* has developed protocols to robustly culture *P. cynomolgi* liver stages in vitro for long periods of time (Dembele et al., 2011; Dembélé et al., 2014)*.* Using the *P. cynomolgi* M strain, we observe equal numbers of *P. cynomolgi* hypnozoites and schizonts in our culture system with the former persisting for more than a month. This has enabled us to screen small molecule inhibitors of liver stage development, leading to the discovery of a histone methyltransferase inhibitor TM2‐115 as an inducer of hypnozoite activation (Dembélé et al., 2014). Although the molecular targets of TM2‐115 in hypnozoites remain uncharacterized, this study indicated that epigenetic regulation of gene expression may underlie hypnozoite quiescence. However, despite the availability of the genome of *P. cynomolgi*—which consists of ~5,700 genes presenting highly conserved synteny with *P. vivax*, but not with *P. falciparum*, distributed on 14 chromosomes (Tachibana et al., 2012)—transcriptomic data are not available for any stage of *P. cynomolgi*, and in the case of *P. vivax,* studies have primarily focused on the blood stage transcriptome (Bozdech et al., 2008; Westenberger et al., 2010; Zhu et al., 2016). We therefore hypothesized that a systematic analysis of the transcriptome of *P. cynomolgi* schizonts and hypnozoites would allow for better understanding of hypnozoite quiescence and commitment. Moreover, such an analysis would support comparative genomic studies that have indicated the existence of *P. cynomolgi*‐ and *P. vivax*‐specific factors that may be involved in this process (Tachibana et al., 2012).

To overcome the challenges of low number and small size of *P. cynomolgi* hypnozoites, we adopted laser capture microdissection (LCM) to isolate these forms. Laser capture microdissection permits direct visualization of heterogeneous cell cultures or tissue sections and subsequent harvesting of specific cell populations for DNA, RNA, and protein profiling (Emmert‐Buck et al., 1996) and has previously been used to study the expression of select liver stage mRNAs of a rodent malaria parasite *P. yoelii* (Sacci et al., 2005) and of the human parasite *P. falciparum* (Semblat, Silvie, Franetich, & Mazier, 2005). LCM is also adaptable to systems in which fluorescently labeled, genetically modified parasites are not readily available. Using LCM technology in simian primary hepatocyte cultures infected with *P. cynomolgi* sporozoites, coupled with protocols for next generation sequencing of cDNA prepared from very low amounts of mRNA, we successfully analyzed the transcriptome of *P. cynomolg*i schizonts and hypnozoites and identified transcription factors of the Apicomplexan AP2 Apetala2 (ApiAP2) family and translational regulators such as the eIF2α kinase, eIK2 as potential regulators of commitment and maintenance of the quiescent state.

## 2 RESULTS AND DISCUSSION

### 2.1 LCM efficiently isolates P. cynomolgi schizonts and hypnozoites

**Figure 1:**
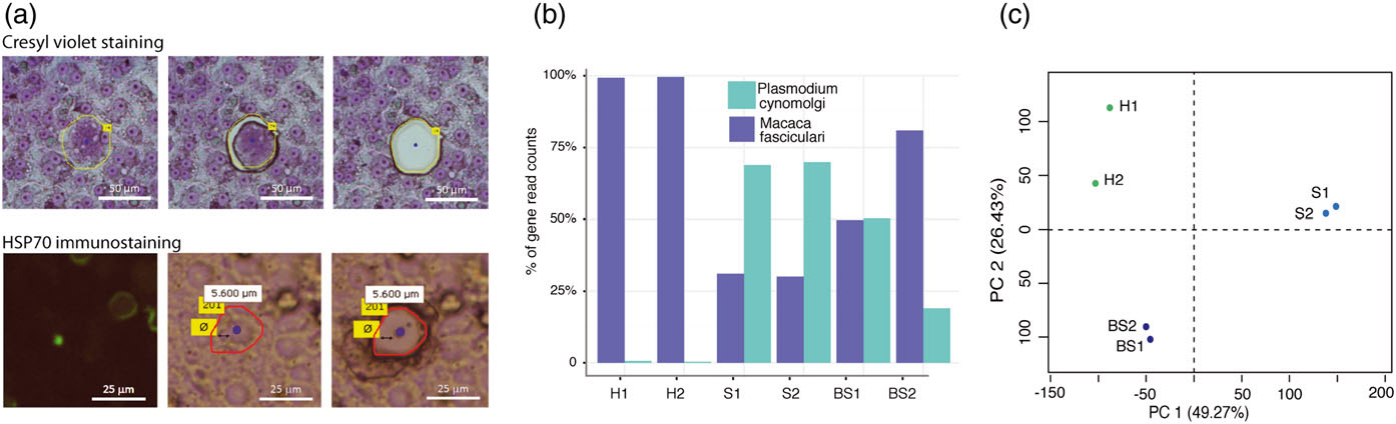
Laser capture microdissection (LCM) efficiently isolates liver stages of *P. cynomolgi* enabling high throughput transcriptomic analyses of these stages. (a) Top: Cresyl violet staining of a *Plasmodium cynomolgi*‐infected monkey hepatocyte culture with a schizont form indicated (left). Bottom: Immunofluorescence microscopy of a *P. cynomolgi*‐infected monkey hepatocyte culture stained with anti‐parasite HSP70 antibodies identifies hypnozoite forms (left). The area selected for LCM is indicated before (middle) and after (right) dissection. (**b)** The percentage of reads that mapped to either the *P. cynomolgi* or the *M. fascicularis* genomes are indicated for each sample. (**d)** position of the samples in the space spanned by the first two components generated from a principal component analysis of the log_2_(read counts) data. **For parts B‐C**, hypnozoite = H1 and H2; liver schizont = S1 and S2; blood stage = BS1 and BS2

To adapt LCM to our system, we cultured M. fascicularis primary hepatocytes on a polyethylene naphthalate membrane ring placed within a 35 mm dish and infected them with *P. cynomolgi* sporozoites isolated from *Anopheles stephensi* mosquitoes (Figure S1a). After 7 days, we detected mature schizonts, which we identified using Cresyl violet staining due to their larger size of around 50 μm diameter, and small hypnozoite forms of 5 μm diameter, which we identified using fixation and immunostaining with anti‐Hsp70 antibodies (Figure 1a). To ensure that RNA from fixed hypnozoites was of suitable quality for downstream processing, we optimized fixation and immunostaining in the following manner: we fixed cells with cold ethanol instead of paraformaldeyde and for immunostaining, utilized higher concentration of antibodies in the presence of RNAse inhibitors and coupled this with short incubation times (Figure S1b). We dissected and pooled 29–69 representative cells of each parasite form (Table S1; 45 hypnozoites for replicate H1 and 59 for replicate H2; 69 schizonts for replicate S1 and 29 for replicate S2) prior to isolating total RNA using silica membrane technology (Figure S1a). Given that the RNA quantities derived from parasite‐infected cells were limited and below detection limit (Table S1), to determine quality, we microdissected a larger area of 5*10^6^ μm^2^, isolated RNA and analyzed it using gel electrophoresis. As shown in Figure S1c, we consistently obtained high quality RNA indicating that neither Cresyl violet staining nor fixation and immunostaining caused RNA degradation.

### 2.2 Next generation sequencing of total RNA from different P. cynomolgi stages

To generate next generation sequencing libraries, we reverse‐transcribed mRNA from microdissected *P. cynomolgi* schizont and hypnozoite samples using the SMARTer Ultra Pico kit, amplified the resulting cDNA using long‐distance polymerase chain reaction, and constructed libraries compatible for Illumina sequencing. As a control and to enable direct comparison to the published *P. vivax* blood stage transcriptome (Bozdech et al., 2008; Westenberger et al., 2010; Zhu et al., 2016), we treated the *P. cynomolgi* blood stage samples that were used to infect *A. stephensi* mosquitoes in a similar manner. The sequencing reads were mapped simultaneously to the *P. cynomolgi* (PCYB v1.0) and *M. fascicularis* (v5.0) genomes using STAR (Figure S1a).

In the case of the liver schizont and blood stage samples, we found that 20–70% of the reads mapped to the *P. cynomolgi* genome and the rest to the *M. fascicularis* genome (Table S1 and Figures 1b and S1b); the *M. fascicularis* reads originate primarily from hepatocytes in the liver schizont sample and from white blood cells in the blood stage sample. In contrast, less than 1% of reads from the hypnozoite samples mapped to the *P. cynomolgi* genome (Table S1 and Figures 1b and S1b). One explanation for this could be that the hypnozoite, being a quiescent cell, has reduced transcriptional activity compared to an actively dividing multinuclear schizont (1,000 to 10,000 transcriptionally active merozoites), and hence reduced mRNA. This has previously been observed for the budding yeast *Saccharomyces cerevisiae,* where global transcriptional shutoff mediated by widespread changes in chromatin structure is associated with its quiescent state (McKnight & Tsukiyama, 2015).

The higher mappability of the *P. cynomolgi* blood stage and liver schizont RNA‐seq libraries resulted in higher coverage with less than 200 genes having zero reads in all of these replicative stages combined (Figure S2a,b; Table S2). In contrast, because of the lower number of reads mapping to the *P. cynomolgi* genome, we observed lower genome‐wide coverage for the hypnozoite sample: more than 2,500 genes did not have any mapped reads in at least one of the two replicates (Figure S2a,b; Table S2). Nonetheless, in all three stages, 50–60% of the reads mapped to annotated *P. cynomolgi* exonic regions with a significant percentage mapping to intergenic regions (Figure S2c). These regions likely correspond to meaningful, unannotated coding or noncoding transcripts that our dataset could help uncover. Of note, the percentage of reads that mapped to genes encoding ribosomal proteins and histones was between 10% and 20% across all samples (Figure S2d). Finally, principal component analysis indicated that the different samples cluster by developmental stage in the space of the first two principal components (Figure 1c), in spite of a lower correlation of 0.4 for gene‐level expression measurements of the two hypnozoite replicates (Figure S2e).

### 2.3 The liver schizont of P. cynomolgi exhibits elevated fatty acid biosynthesis activity relative to the blood stage

Thus far, the only transcriptomic data available for malaria parasite liver stages is for the rodent malaria parasite *P. yoelii* using microarrays (Tarun et al., 2008)*,* which revealed the expression of 2,000 mRNAs in liver schizonts and upregulation of a key metabolic pathway involved in lipid biosynthesis, the Fatty Acid Synthesis II pathway. In our study, we identified a larger cohort of mRNAs expressed in the two liver schizont replicates—4,598 out of the 5,776 annotated *P. cynomolgi* genes had at least 1 count per million (CPM) in the two replicates (Figures S3a and 2a; Table S2). Moreover, we obtained high quality data from a single *P. cynomolgi* liver schizont (Figure S3) in which 3,213 genes had at least 1 CPM, and correlated well to the transcriptome of the pooled samples S1 and S2 (Pearson correlation coefficient of .65; Figure S3b). Together, this emphasizes the potential of the LCM technique to perform single cell RNA‐seq of proliferative liver stages.

**Figure 2:**
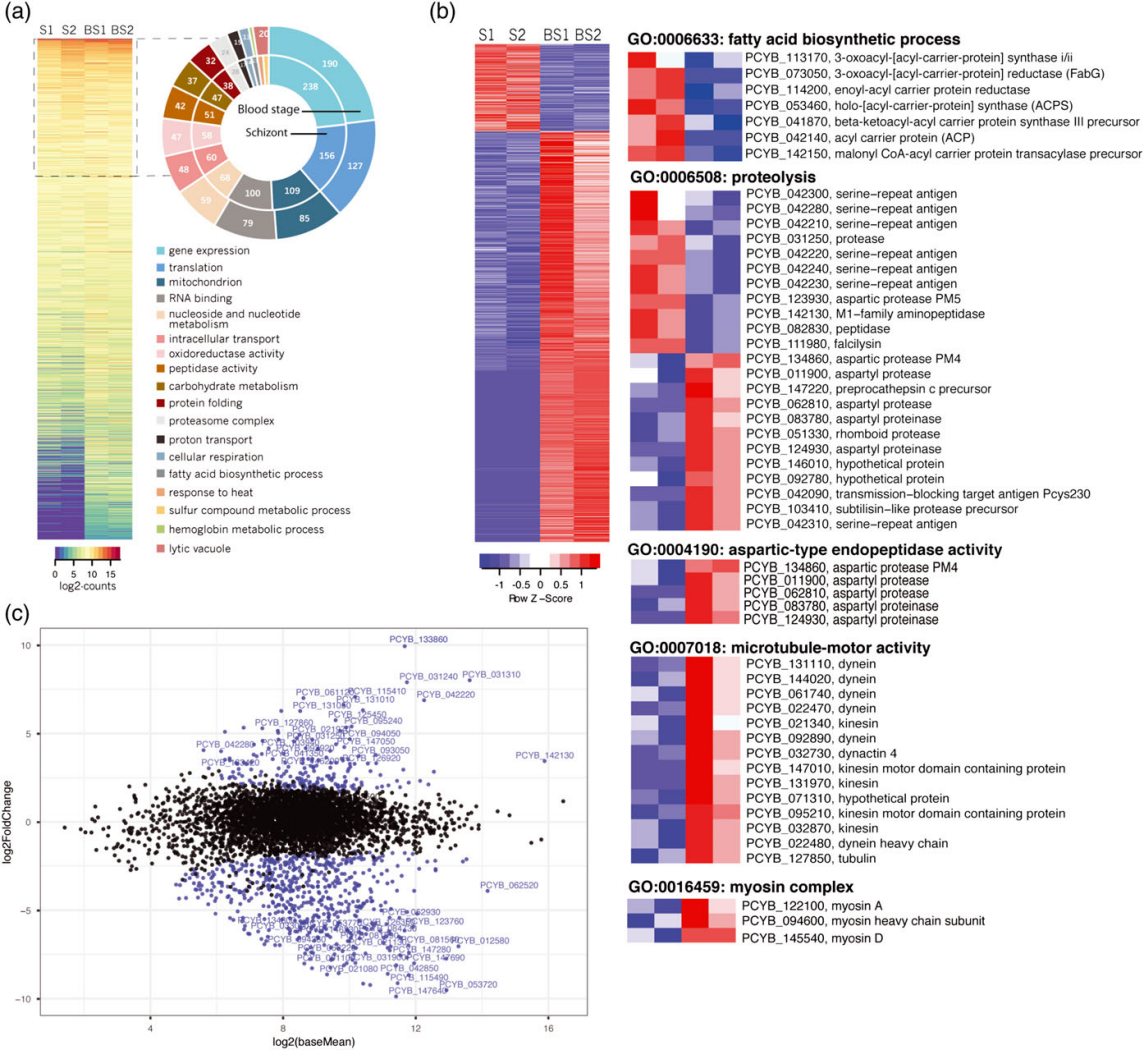
The *P. cynomolgi* liver schizont transcriptome is enriched with mRNAs encoding components of the fatty acid biosynthesis pathway. (a) Heat map showing the gene expression (log_2_normalized‐read‐counts) of 4,801 genes with at least 1 CPM in at least two of the four schizont (S) and blood stage (BS) samples. Gene ontology (GO) terms enriched in genes with high expression (in the top 25% of expression levels) are represented in a doughnut graph to the right of the heat map. (**b)** Heat map showing the gene expression (log_2_normalized‐read‐counts centered and scaled for each row) of the 807 genes detected as differentially expressed between the schizont (S) and blood stage (BS) samples. Zooming in, specific GO terms of interest enriched within the list of differentially expressed genes are shown to the right of the heat map. (**c)** The mean expression of genes in the schizont and blood stage datasets (x‐axis) was compared to the log_2_(fold change) of gene expression in schizonts relative to the blood stages (y‐axis) and represented as an MA‐plot using the DESeq2 package. Genes with an FDR < 5% are represented with a blue dot and the names of those with a log_2_(fold change) >2 are shown

To analyze the properties of the liver schizont transcriptome, we performed Gene Ontology (GO) analysis of genes expressed at different levels within individual samples using GO terms associated with the syntenic *P. vivax* or *P. falciparum* ortholog of each gene. We identified pathways such as gene expression, translation, RNA‐binding, carbohydrate metabolism, nucleotide metabolism, and intracellular transport as being enriched in the top 25th percentile of expression (Figure 2a). When compared to the top 25th percentile of the *P. cynomolgi* blood stage transcriptome—5,522 expressed genes with at least 1 CPM and 4,648 genes with at least 10 CPM in both replicates (Figure S4a and Table S2)—pathways such as response to heat stress, fatty acid biosynthesis, and sulphur compound biosynthesis were over‐represented (Figure 2a; see doughnut chart). In contrast, the top 25th percentile of the blood stage transcriptome was enriched for GO terms such as hemoglobin metabolism and digestive vacuole (Figure 2a; see doughnut chart). Given that our blood stage datasets correlated to patient samples CM012 (63% asexual and 37% gametocyte stages) and CM013 (83% asexual stages and 11% gametocytes) from Westenberger et al. microarray analysis of the *P. vivax* transcriptome (Westenberger et al., 2010) with a Pearson correlation coefficient of .5 (Figure S4b), our analysis supports the use of *P. cynomolgi* transcriptomic analysis to obtain insights into *P. vivax* biology.

To further contrast the metabolic state of the dividing liver and blood stage forms of *P. cynomolgi*, we performed differential expression analysis using DESeq2 (Love, Huber, & Anders, 2014) of 4,801 genes detected at ≥1 CPM in at least one of the two samples of each stage (Figure S5a and Table S3). We identified 807 genes as differentially expressed (DEX; FDR <5%), 212 of which were >3‐fold upregulated and 595 of which were >3‐fold downregulated in the schizont samples (Figure 2b,c and Table S3). GO enrichment analysis showed that upregulated schizont transcripts are enriched for pathways such as fatty acid biosynthesis, cysteine‐type peptidase activity, and endopeptidase activity (Figure 2b and Table S3). Moreover, the only enriched GO term in the cellular component category is the apicoplast (Table S3), and is associated with genes involved in fatty acid biosynthesis: this in keeping with studies in *P. yoelii*, which suggested that the Fatty Acid Synthesis II pathway is an important component of liver development (Tarun et al., 2008). On the other hand, the downregulated genes are enriched in GO terms related to structural constituents of the cytoskeleton and microtubule motor activity (Figure 2b), implying that microtubule motor components may play a key role in blood stage development, but not in liver schizont development. Notably, we found that the mRNAs of Liver Specific Protein 1 and 2 (LISP1 and LISP2) and sporozoite and liver stage asparagine‐rich proteins (SLARP), proteins involved in parasite egress (LISP1; (Ishino et al., 2009)), host–parasite interaction (LISP2; (Orito et al., 2013)) and initiation of liver stage development (SLARP; [Silvie, Goetz, & Matuschewski, 2008]) were upregulated specifically in the *P. cynomolgi* liver schizont. Overall, our data demonstrate that *P. cynomolgi* upregulates different *Plasmodium*‐conserved pathways to divide within the liver cell as compared to a red blood cell, confirming the existence of host cell type‐specific adaptations of gene expression.

### 2.4 The first description of the hypnozoite transcriptome

**Figure 3:**
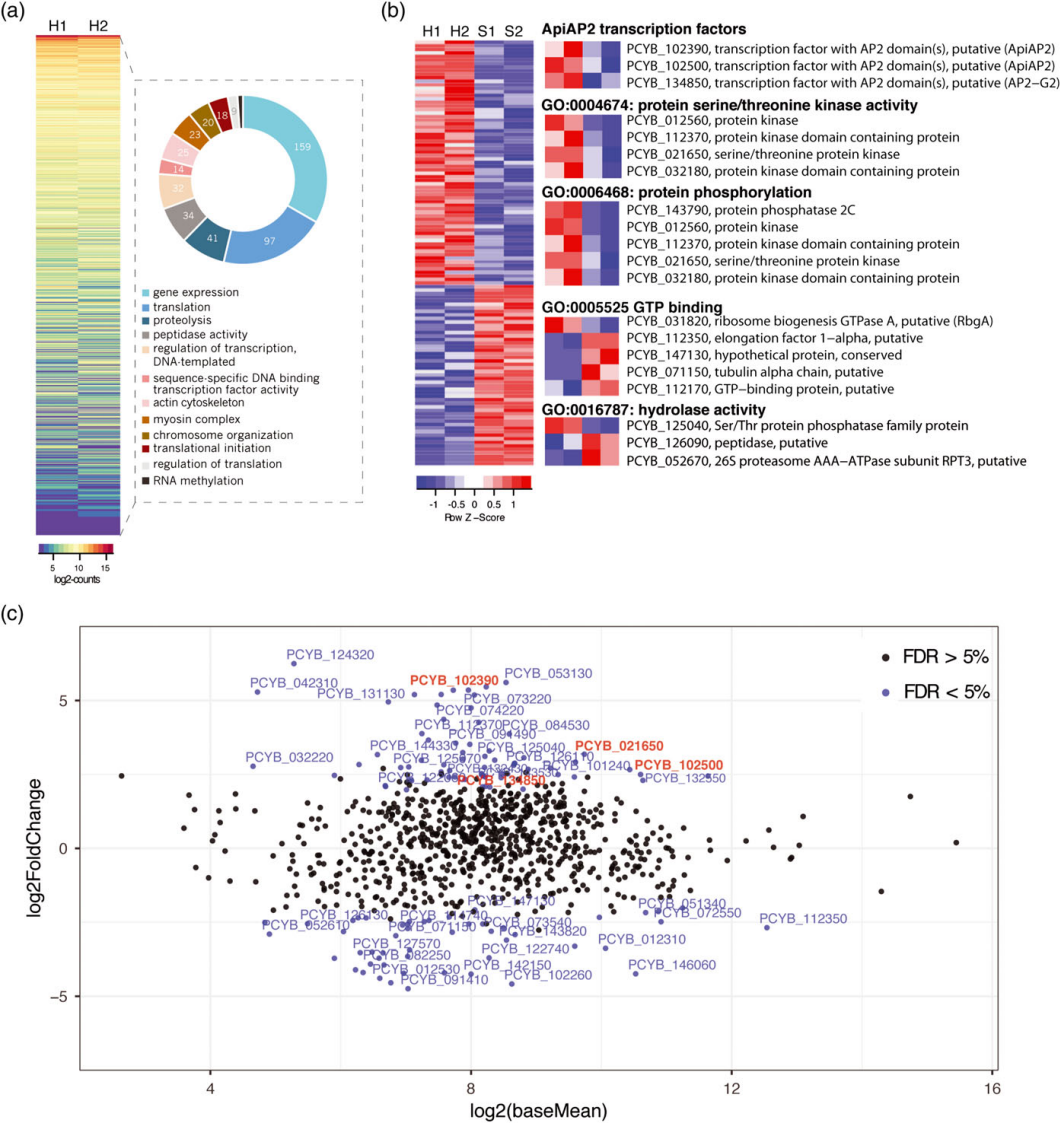
The quiescent state of the *P. cynomolgi* hypnozoite may be regulated by transcription factors of the ApiAP2 family and translational repression by the eIF2a kinase eIK2. (a) Heat map showing the gene expression (log_2_normalized‐read‐counts) of 880 genes with at least one read count in both hypnozoite (H) replicates. Gene ontology (GO) terms enriched in all 880 genes are represented in a doughnut graph to the right of the heat map. (**b)** Heat map showing the gene expression (log_2_normalized‐read‐counts centered and scaled for each row) of the 120 genes detected as differentially expressed between the hypnozoite (H) and schizont (S) samples. Zooming in, specific GO terms of interest enriched within the list of differentially expressed genes are shown to the right of the heat map. **(c)** The mean expression of genes in the hypnozoite and schizont stage datasets (x‐axis) was compared to the log_2_(fold change) of gene expression in hypnozoites relative to schizonts (y‐axis) and represented as an MA‐plot using DESeq2. Genes with an FDR < 5% are represented with a blue dot and the names of those with a log_2_(fold change) >2 are shown. Genes of interest are highlighted in red

Due to the small fraction of sequencing reads that mapped to the *P. cynomolgi* genome (Figure 1b and Table S1) in the hypnozoite samples, only 2,464 genes had at least one read count in one of the two hypnozoite samples (Table S2). Low read counts together with a limited number of replicates are likely to increase variation, affect type I errors and estimation accuracy of the differential expression model. Hence, we proceeded cautiously and selected 880 genes with at least 1 read count in both hypnozoite replicates as the most representative of this quiescent state (Table S4). GO analysis with *P. vivax* or *P. falciparum* syntenic orthologs of these genes provided a generalized view of the hypnozoite transcriptome: pathways such as gene expression, translation, proteolysis, and chromosome organization, are enriched (Figure 3a). This list is similar to the top 25th percentile of the liver schizont and blood stage transcripts (Figure 2a) except for the presence of the GO term “regulation of transcription, DNA‐templated,” which is associated with 32 genes involved in negative regulation of transcription, chromatin remodeling, chromatin silencing at telomeres, and protein ubiquitination, all of which could play a role in global transcriptional repression, partially explaining the reduced number of *P. cynomolgi* mRNA reads in the quiescent state.

To identify potential biomarkers of the hypnozoite, we compared the hypnozoite and liver schizont transcriptome using DESeq2 (Figure S5b) and detected 120 genes as being DEX (FDR <5%): 69 > 3‐fold upregulated and 51 > 3‐fold downregulated in the hypnozoite samples (Table S4 and Figure 3b,c). Enriched GO terms include protein serine or threonine kinase activity and methyltransferase activity for the upregulated genes, and guanosine 5'‐triphosphate binding, hydrolase activity, and threonine‐type endopeptidase activity for the downregulated genes (Table S4 and Figure 3b). Notably, this list contains 12 genes that are absent in *P. falciparum* (Table S5), 11 of which have orthologs in *P. vivax* and *P. ovale* and one is specific to *P. cynomolgi* and belongs to the variant Pv‐fam‐e/RAD family. Moreover, the genes that were predicted to play a role in hypnozoite formation by Tachibana et al. in the *P. cynomolgi* genome assembly study (Tachibana et al., 2012), including the Ran guanosine 5'‐triphosphate‐binding protein *PCYB_092380*, did not have any reads in the hypnozoite sample neither did hypnozoite regulatory genes predicted by the *P. ovale* genome assembly study (Rutledge et al., 2017; Table S5). We discuss the putative role of some of the DEX factors in hypnozoite commitment and maintenance in the next sections, focusing on transcripts that have at least 10 read counts in at least one of the replicates.

### 2.5 ApiAP2 transcription factors as potential regulators of hypnozoite commitment and/or maintenance

The 27‐member ApiAP2 family of specialized transcription factors is homologous to the plant Apetala2/Ethylene response factor family and is characterized by one or more 60 aa DNA‐binding AP2 domains (Painter, Campbell, & Llinás, 2011). In recent years, the role of the ApiAP2 proteins in determining stage transitions during the *Plasmodium spp.* life cycle has become evident (Kafsack et al., 2014; Modrzynska et al., 2017; Sinha et al., 2014). Intriguingly, in *P. cynomolgi* hypnozoites, we observed that three ApiAP2 genes are upregulated (Figure 3b,c), *PCYB_102500*, *PCYB_102390*, and *PCYB_134850* (FDR = 0.053; Table S4). Of these, *PCYB_102500* and *PCYB_134850* present orthologs in all other *Plasmodium spp.* while orthologs of *PCYB_102390* are identifiable only in *P. vivax* and *P. ovale*. This indicates that the *PCYB_102390*‐encoded ApiAP2, which we term AP2‐Q (for quiescence), might be responsible for commitment to quiescence, as has been observed for a second transcriptional activating ApiAP2, AP2‐G, during sexual commitment (Kafsack et al., 2014; Sinha et al., 2014). Indeed, AP2‐Q could be the first biomarker of hypnozoites and further studies are needed to confirm the role of this protein in hypnozoite transcriptional regulation.

While a recent study predicted a role for *PCYB_102500* orthologs in transcriptional regulation in transmission stages (so‐called AP2‐O2 for a phenotype in mosquito ookinete development; (Modrzynska et al., 2017), the ortholog of *PCYB_134850* in the rodent malaria parasite *P. berghei* encodes for AP2‐G2, described as a second regulator of gametocyte commitment, downstream of AP2‐G action (Sinha et al., 2014; Yuda, Iwanaga, Kaneko, & Kato, 2015). PbAP2‐G2 transcriptionally represses around 1,500 asexual stage genes, thus blocking the asexual proliferation program of sexually committed parasites (Yuda et al., 2015). When we scanned the upstream promoter regions (1–1.5 kb region upstream of transcription start site or ATG) of the DEX genes for the recognition motif of AP2‐O2 (TGATATCA) or AP2‐G2 ([A/T/G]GTTG[T/C][A/T/C] and other variations of the same), we detected their occurrence in 25–98% of the hypnozoite stage DEX genes (Table S6). However, we did not observe a specific enrichment of these motifs in DEX genes relative to non‐DEX genes (Table S6). Furthermore, motif enrichment analysis using MEME (Bailey et al., 2009) did not identify a specific sequence in the upstream promoter regions of the DEX genes that could be targeted for transcriptional regulation.

### 2.6 Similarity to other nondividing states of Plasmodium and non‐Plasmodium spp.

A well‐characterized quiescent state of malaria parasites is the mature salivary gland sporozoite, with low levels of transcription and translational repression playing a key role in maintaining its metabolic state (Zhang et al., 2010). To determine if similar processes regulate the hypnozoite, we first compared the hypnozoite transcriptome to the *P. vivax* sporozoite transcriptome (Westenberger et al., 2010) and found that only 11 of the top 50 highly expressed sporozoite genes were also expressed by the *P. cynomolgi* hypnozoite (highlighted in red in Table S4). Second, given that sporozoite translational repression is mediated globally by the eukaryotic initiation factor‐2α kinase eIK2, a serine or threonine kinase (Zhang et al., 2010), and specifically by RNA‐binding proteins of the Pumilio and fem‐3 binding factor or Puf family, Puf1 and Puf2 (Gomes‐Santos et al., 2011; Miao et al., 2013), we scanned the list of DEX genes and identified a serine or threonine protein kinase (encoded by *PCYB_021650*) as being upregulated in the hypnozoite transcriptome (Figure 3b,c and Table S4) and presenting an ortholog only in *P. vivax* (Table S5) and *P. ovale*. Using Blastp on https://plasmodb.org with the *PCYB_021650*‐encoded protein, we selected the top 50 hits, phylogenetic analysis of which classified *PCYB_021650* as a potential *P. cynomolgi* eIK2 ortholog (Figure S6). The upregulation of eIK2 in *P. cynomolgi* hypnozoites suggests that translation repression could partially contribute to maintenance of the quiescent state, although we cannot rule out other functions for *PCYB_021650*. We also identified the mRNAs of other RNA‐binding proteins including *PCYB_094480* (contains an RNA recognition motif) and *PCYB_053300* (NOB1‐like protein) as being upregulated in the *P. cynomolgi* hypnozoite, indicating that posttranscriptional gene regulation may also contribute to hypnozoite homeostasis.

Eukaryotic cells such as mammalian cells, budding yeast cells, and dividing *Toxoplasma gondii* tachyzoites, differentiate into a quiescent state under conditions of stress or starvation or in response to external stimuli. In the case of mammalian cells, the G0 phase of the cell cycle is a classic resting state, and several GO terms have been associated with cell cycle arrest or G0 entry (http://amigo.geneontology.org/amigo), none of which are enriched in the hypnozoite genes. Nonetheless, cell cycle regulators, *PCYB_063140* (enhancer of rudimentary domain containing protein), *PCYB_102940* (CDK‐activating kinase assembly factor), *PCYB_146400* (cell cycle control protein), *PCYB_133040* (proliferation‐associated protein 2 g4), *PCYB_126660*(SEL‐1 protein), and others are expressed by the hypnozoite and may effect quiescence. In the case of *S. cerevisiae*, 15 genes—CTA1, ARP4, CTT1, VRG4, RIM15, GVP36, TOR1, VAN1, SCH9, ARD1, SOD2, SPA2, SIC1, HOG1, and SNF1—are associated with entry into G0 (http://www.yeastgenome.org/), and are enriched for GO terms such as “response to stress” and “protein phosphorylation,” both of which are enriched within the 880 hypnozoite genes selected for our analysis. Finally, for *T. gondii* tachyzoites, differentiation into the latent bradyzoite state is associated with the expression of over 100 factors (Buchholz et al., 2011); orthologs of several of these factors are expressed in the hypnozoites (Table S7) and present as important candidates to explore the biology of this quiescent state. Indeed, this list contains genes encoding RNA‐binding proteins (*PCYB_112050*), high mobility group proteins (*PCYB_052380* and *PCYB_131170*), translation initiation factors (*PCYB_146820*), enolase (*PCYB_082570*), and lactate dehydrogenase (*PCYB_123790*), all of which are of potential interest to elucidating hypnozoite biology.

### 2.7 Conclusions and perspectives

Much research has gone into developing drugs that target all stages of *Plasmodium* growth, including the inaccessible hypnozoite form, with the only one FDA‐approved that kills this quiescent stage being primaquine (Hill et al., 2006). However, primaquine and its derivatives suffer from the caveat that they cause haemolysis in people with glucose‐6‐phosphate dehydrogenase deficiencies, a genetic trait prevalent in the populations where *P. vivax* malaria is endemic, and are ineffective in people with low metabolising cytochrome P450 2D6 genotypes (Campo, Vandal, Wesche, & Burrows, 2015). Therefore, there is an urgent need to discover novel antihypnozoite compounds that can be widely deployed for the treatment and radical cure and/or prophylaxis of *P. vivax* and *P. ovale* malaria. To our knowledge, our study is the first to characterize the hypnozoite transcriptome of any malaria‐relapsing species identifying key factors that regulate transcription and translation, pathways that permit a cell to proliferate, as potential markers of the hypnozoite. Further studies on genes highlighted in this study could help to clarify the properties of the quiescent state and identify ways to wake up and kill these dormant forms.

## 3 EXPERIMENTAL PROCEDURES

### 3.1 Ethics statement

All studies that involved animals are in compliance with the standards for human care and use of laboratory animals (Animal welfare Assurance, OLAW number A5826‐01) and are in accordance with French national regulation as well as European guidelines. See supplementary information for additional details.

### 3.2 Hepatocyte infection with P. cynomolgi sporozoites

*P. cynomolgi* M strain sporozoites and *M. fascicularis* hepatocytes were cultured as previously described (Dembele et al., 2011; Dembélé et al., 2014); see supplementary information for additional details). For liver stage *P. cynomolgi* infection in an LCM‐compatible setup, *M. fascicularis* hepatocytes were seeded at a final cell density of 250,000 cells per cm^2^ in a polyethylene naphthalate ‐membrane Ring 35 (Zeiss) coated with collagen I (BD BioSciences) in a lummox dish support (Zeiss). 6 × 10^5^ *P. cynomolgi* sporozoites resuspended in complete medium were then added to the hepatocyte culture and incubated at room temperature for 3 hr to allow parasite sedimentation and at 37°C with 5% CO_2_ for 3 hr to allow hepatocyte invasion and infection. After this, free sporozoites were washed away and the culture incubated at 37°C with 5% CO_2_ for up to 7 days. The medium was changed every 24 to 48 hr.

### 3.3 Laser capture microdissection

*P. cynomolgi*‐infected hepatocytes were microdissected at day 7 postinfection. The schizont forms were visualized using a modified Cresyl violet (Sigma) staining protocol and hypnozoites were visualized using a rapid immunostaining method optimized for RNA preservation (Figure S1b). See supplementary information for additional details. LCM was carried out using the PALM microlaser system (Zeiss) and the PALM‐Robo software, which permits the selection, posterior cut and catapulting of regions of interest into a collection tube. 1, 29 or 69 schizont stage parasites and 45 or 59 hypnozoites were captured and pooled for RNA preparation as described below.

### 3.4 RNA extraction and next generation sequencing

Microdissected schizonts and hypnozoites were directly suspended in RA1 buffer complemented with TCEP as a reducing agent (Machery‐Nagel) and total RNA extracted using the NucleoSpin RNA XS kit (Macherey‐Nagel). In the case of *P. cynomolgi* blood stage cultures, RNA was extracted from frozen infected *M. fascicularis* blood samples. Briefly, the blood samples were centrifugated for 10 min at room temperature at 500 g and washed with PBS prior to RNA extraction using the NucleoSpin RNA XS kit. RNA quality was assessed on an Agilent Bioanalyzer 2100 using the RNA Pico kit (Agilent Biotechnologies). Total RNA profiles with RNA integrity number superior to 8 were considered for downstream analysis. cDNA synthesis and amplification using long‐distance polymerase chain reaction was performed using the SMART‐Seq v4 Ultra Low Input RNA Kit (Clontech; Takara). The resulting cDNA was assessed for quality using the Bionanalyzer 2100 and DNA HS kit. Illumina‐compatible sequencing libraries were prepared using the Nextera XT DNA Library Preparation Kit (Illumina), multiplexed and sequenced using a single read 150‐nucleotide (SR150) run on the NextSeq 500 sequencer (Illumina). A minimum of two biological replicates was analyzed for each sample type (Table S1).

### 3.5 Bioinformatic analysis

The *P. cynomolgi* genome sequence (v1.0, version 28) and gene annotations were downloaded from PlasmoDB (https://plasmodb.org). The *M. fascicularis* genome sequence was downloaded from National Center for Biotechnology Information, annotation release 101 (https://www.ncbi.nlm.nih.gov/genome/776). Sequencing reads were mapped simultaneously to the *P. cynomolgi* and the *M. fascicularis* genomes with STAR v2.5.0b (Dobin et al., 2013) using default parameters. Mapped reads were counted for each annotated genomic feature with the FeatureCount read summarization program from the Subread package (v1.4.6) (Liao, Smyth, & Shi, 2014). To study the distribution of the reads across features, the read_distribution function of the RSeQC (v2.6.4) package (Wang, Wang, & Li, 2012) was used. Differentially expressed genes were identified using DESeq2 v1.14.1 with default parameters. For GO analysis, the orthologs of *P. vivax* and *P. falciparum* were identified using PlasmoDB and annotated or predicted GO terms extracted. Enrichment of GO categories for genes in the top 25th percentile of expression in the schizont and blood stage samples and the 880 genes expressed in the hypnozoites was evaluated using PlasmoDB and for the differentially expressed genes using the Bioconductor package GOseq (v1.24.0) (Young, Wakefield, Smyth, & Oshlack, 2010).

## AUTHOR CONTRIBUTIONS

R.C., S.S.V., A.S., D.M. designed research; R.C., S.S.V., J.F.F., M.B., D.S., G.Z., H.V., M.B., A.M., N.D.B., R.L.G. performed research; R.C., S.S.V., A.B analyzed data; and S.S.V., R.C. and A.B. wrote the paper.

The authors declare no conflict of interest.

## AVAILABILITY OF DATA

All fastq files generated in this study are available in the EMBL‐EBI European Nucleotide Archive [ENA: PRJEB18141; Sample group: ERS1461774]: http://www.ebi.ac.uk/ena/data/view/PRJEB18141

## Supporting information

Data S1. Supporting info item

**Figure S1:** **A.** Schematic representation of the different steps of the Laser Capture Microdissection (LCM) and RNA‐seq library preparation protocol for *Plasmodium cynomolgi* liver stages. **B.** Optimized LCM‐adapted Cresyl violet staining protocol for the detection of liver schizonts (upper panel) and immunostaining protocol for the detection of hypnozoites (lower panel). **C.** Representative electropherogram of RNA extracted from a control microdissected area for the schizont (upper panel) and hypnozoite (lower panel) samples showing the RNA Integrity Number (RIN). Samples with a RIN > 8 were chosen for RNA‐seq library preparation.

**Figure S2:** **A.** Density distribution of the read counts for the four samples from dividing stages (Schizont “S” and Blood stage “BS”, left panel) and the two biological replicates of the non‐dividing hypnozoite stage (“H”, right panel). **B.** Venn diagram showing the number of genes with at least 1 read count per million (CPM) in the two biological replicates of each stage and the overlap in genes with at least 1 CPM in all samples. **C.** Distribution of reads across annotated regions of the *P. cynomolgi* genome. **D.** Percentage of reads mapping to *P. cynomolgi* ribosomal or histone genes. **E.** Scatter plots showing all pairwise log_2_CPM correlations between the six samples. The upper right part of the panel shows the value of the calculated Pearson correlation coefficients.

**Figure S3:** **A.** Venn diagram showing the number of genes with at least 1 CPM in the two biological replicates of the *P. cynomolgi* liver schizont (pooled samples S1 and S2) and the single liver schizont. **B.** Scatter plots showing all pairwise log_2_CPM correlations between S1, S2 and the single schizont sample. The upper right part of the panel shows the value of the calculated Pearson correlation coefficients.

**Figure S4:** **A.** Venn diagram showing the number of genes with at least 1 CPM (left) or 10 CPM (right) in the two biological replicates of the *P. cynomolgi* blood stage samples. **B.** Comparison of the gene expression between *P. cynomolgi* blood stages (average log_2_(read counts +1) of the two replicates; x‐axis) and the microarray data from Westenberger *et al.* (ref) for *P. vivax* blood stages (average of samples CM012 and CM013; y = axis).

**Figure S5:** Plots of the DESeq2 per‐gene dispersion estimates together with the fitted mean‐dispersion relationship for: **A.** The 4801 genes selected for the Schizont vs Blood Stage comparison, and B. The 880 genes selected for the Hypnozoite and Schizont comparison.

**Figure S6:** Phylogenetic tree of the top 50 *P. falciparum, P. vivax, P. berghei* and *P. cynomolgi* hits obtained from Blastp analysis of serine/threonine kinase encoded by the *PCYB_021650*. Highlighted in red is the eIF2α kinase eIK2 group containing *PCYB_021650* and its putative *P. falciparum*, *P. vivax* and *P. berghei* orthologs. The tree was constructed using the Treedyn phylogeny representation software.

Table S1: Total number of reads that mapped to the P. cynomolgi genome in the hypnozoite, liver schizont and blood stage samples.

Table S2: Raw read counts of different *Plasmodium cynomolgi* annotated genes.

Table S3 C: Gene Ontology analysis of schizont downregulated genes.

Table S4 A: Differential expression (DEX) analysis of the transcriptomes of hypnozoite vs schizonts.

Table S5: List of putative factors involved in hypnozoite dormancy and their expression levels in the different samples.

Table S6: Detection of AP2‐G2 and AP2‐O2 motifs in the upstream promoter region of hypnozoite DEX genes.

Table S7 List of *P. cynomolgi* orthologs of factors involved in bradyzoite latency and their expression levels in the different samples.

## References

[cmi12735-bib-0001] Bailey, T. L. , Boden, M. , Buske, F. A. , Frith, M. , Grant, C. E. , Clementi, L. , … Noble, W. S. (2009). MEME SUITE: Tools for motif discovery and searching. Nucleic Acids Research, 37, W202–W208. 19458158 10.1093/nar/gkp335 PMC2703892

[cmi12735-bib-0002] Bozdech, Z. , Mok, S. , Hu, G. , Imwong, M. , Jaidee, A. , Russell, B. , … Preiser, P. R. (2008). The transcriptome of *Plasmodium vivax* reveals divergence and diversity of transcriptional regulation in malaria parasites. Proceedings of the National Academy of Sciences of the United States of America, 105, 16290–16295. 18852452 10.1073/pnas.0807404105 PMC2571024

[cmi12735-bib-0003] Buchholz, K. R. , Fritz, H. M. , Chen, X. , Durbin‐Johnson, B. , Rocke, D. M. , Ferguson, D. J. , … Boothroyd, J. C. (2011). Identification of tissue cyst wall components by transcriptome analysis of in vivo and in vitro Toxoplasma gondii bradyzoites. Eukaryotic Cell, 10, 1637–1647. 22021236 10.1128/EC.05182-11 PMC3232729

[cmi12735-bib-0004] Campo, B. , Vandal, O. , Wesche, D. L. , & Burrows, J. N. (2015). Killing the hypnozoite‐‐drug discovery approaches to prevent relapse in *Plasmodium vivax* . Pathogens and Global Health, 109, 107–122. 25891812 10.1179/2047773215Y.0000000013 PMC4455353

[cmi12735-bib-0005] Dembélé, L. , Franetich, J. F. , Lorthiois, A. , Gego, A. , Zeeman, A. M. , Kocken, C. H. , … Mazier, D. (2014). Persistence and activation of malaria hypnozoites in long‐term primary hepatocyte cultures. Nature Medicine, 20, 307–312. 10.1038/nm.3461 24509527

[cmi12735-bib-0006] Dembele, L. , Gego, A. , Zeeman, A. M. , Franetich, J. F. , Silvie, O. , Rametti, A. , … Mazier, D. (2011). Towards an in vitro model of Plasmodium hypnozoites suitable for drug discovery. PloS One, 6, e18162. 21483865 10.1371/journal.pone.0018162 PMC3069045

[cmi12735-bib-0007] Dobin, A. , Davis, C. A. , Schlesinger, F. , Drenkow, J. , Zaleski, C. , Jha, S. , … Gingeras, T. R. (2013). STAR: Ultrafast universal RNA‐seq aligner. Bioinformatics, 29, 15–21. 23104886 10.1093/bioinformatics/bts635 PMC3530905

[cmi12735-bib-0008] Emmert‐Buck, M. R. , Bonner, R. F. , Smith, P. D. , Chuaqui, R. F. , Zhuang, Z. , Goldstein, S. R. , … Liotta, L. A. (1996). Laser capture microdissection. Science, 274, 998–1001. 8875945 10.1126/science.274.5289.998

[cmi12735-bib-0009] Galinski, M. R. , Meyer, E. V. , & Barnwell, J. W. (2013). Plasmodium vivax: Modern strategies to study a persistent parasite's life cycle. Advances in Parasitology, 81, 1–26. 23384620 10.1016/B978-0-12-407826-0.00001-1

[cmi12735-bib-0010] Gomes‐Santos, C. S. , Braks, J. , Prudêncio, M. , Carret, C. , Gomes, A. R. , Pain, A. , … Mota, M. M. (2011). Transition of Plasmodium sporozoites into liver stage‐like forms is regulated by the RNA binding protein Pumilio. PLoS Pathogens, 7, e1002046. 21625527 10.1371/journal.ppat.1002046 PMC3098293

[cmi12735-bib-0011] Hill, D. R. , Baird, J. K. , Parise, M. E. , Lewis, L. S. , Ryan, E. T. , & Magill, A. J. (2006). Primaquine: Report from CDC expert meeting on malaria chemoprophylaxis I. The American Journal of Tropical Medicine and Hygiene, 75, 402–415. 16968913

[cmi12735-bib-0012] Ishino, T. , Boisson, B. , Orito, Y. , Lacroix, C. , Bischoff, E. , Loussert, C. , … Baldacci, P. (2009). LISP1 is important for the egress of Plasmodium berghei parasites from liver cells. Cellular Microbiology, 11, 1329–1339. 19438514 10.1111/j.1462-5822.2009.01333.x PMC2774474

[cmi12735-bib-0013] Kafsack, B. F. , Rovira‐Graells, N. , Clark, T. G. , Bancells, C. , Crowley, V. M. , Campino, S. G. , … Llinás, M. (2014). A transcriptional switch underlies commitment to sexual development in malaria parasites. Nature, 507, 248–252. 24572369 10.1038/nature12920 PMC4040541

[cmi12735-bib-0014] Liao, Y. , Smyth, G. K. , & Shi, W. (2014). featureCounts: An efficient general purpose program for assigning sequence reads to genomic features. Bioinformatics, 30, 923–930. 24227677 10.1093/bioinformatics/btt656

[cmi12735-bib-0015] Love, M. I. , Huber, W. , & Anders, S. (2014). Moderated estimation of fold change and dispersion for RNA‐seq data with DESeq2. Genome Biology, 15, 550. 25516281 10.1186/s13059-014-0550-8 PMC4302049

[cmi12735-bib-0016] Mazier, D. , Landau, I. , Druilhe, P. , Miltgen, F. , Guguen‐Guillouzo, C. , Baccam, D. , … Gentilini, M. (1984). Cultivation of the liver forms of Plasmodium vivax in human hepatocytes. Nature, 307, 367–369. 6363939 10.1038/307367a0

[cmi12735-bib-0017] McKnight, J. N. , & Tsukiyama, T. (2015). The conserved HDAC Rpd3 drives transcriptional quiescence in S. cerevisiae . Genomics Data, 6, 245–248. 26697386 10.1016/j.gdata.2015.10.008 PMC4664762

[cmi12735-bib-0018] Miao, J. , Fan, Q. , Parker, D. , Li, X. , Li, J. , & Cui, L. (2013). Puf mediates translation repression of transmission‐blocking vaccine candidates in malaria parasites. PLoS Pathogens, 9, e1003268. 23637595 10.1371/journal.ppat.1003268 PMC3630172

[cmi12735-bib-0019] Modrzynska, K. , Pfander, C. , Chappell, L. , Yu, L. , Suarez, C. , Dundas, K. , … Billker, O. (2017). A knockout screen of ApiAP2 genes reveals networks of interacting transcriptional regulators controlling the Plasmodium life cycle. Cell Host & Microbe, 21, 11–22. 28081440 10.1016/j.chom.2016.12.003 PMC5241200

[cmi12735-bib-0020] Orito, Y. , Ishino, T. , Iwanaga, S. , Kaneko, I. , Kato, T. , Menard, R. , … Yuda, M. (2013). Liver‐specific protein 2: A Plasmodium protein exported to the hepatocyte cytoplasm and required for merozoite formation. Molecular Microbiology, 87, 66–79. 23216750 10.1111/mmi.12083

[cmi12735-bib-0021] Painter, H. J. , Campbell, T. L. , & Llinás, M. (2011). The Apicomplexan AP2 family: Integral factors regulating Plasmodium development. Molecular and Biochemical Parasitology, 176, 1–7. 21126543 10.1016/j.molbiopara.2010.11.014 PMC3026892

[cmi12735-bib-0022] Rutledge, G. G. , Böhme, U. , Sanders, M. , Reid, A. J. , Cotton, J. A. , Maiga‐Ascofare, O. , … Otto, T. D. (2017). Plasmodium malariae and P. ovale genomes provide insights into malaria parasite evolution. Nature, 542, 101–104. 28117441 10.1038/nature21038 PMC5326575

[cmi12735-bib-0023] Sacci, J. B. , Ribeiro, J. M. , Huang, F. , Alam, U. , Russell, J. A. , Blair, P. L. , … Aguiar, J. C. (2005). Transcriptional analysis of in vivo Plasmodium yoelii liver stage gene expression. Molecular and Biochemical Parasitology, 142, 177–183. 15876462 10.1016/j.molbiopara.2005.03.018

[cmi12735-bib-0024] Semblat, J. P. , Silvie, O. , Franetich, J. F. , & Mazier, D. (2005). Laser capture microdissection of hepatic stages of the human parasite Plasmodium falciparum for molecular analysis. Methods in Molecular Biology, 293, 301–307. 16028429 10.1385/1-59259-853-6:301

[cmi12735-bib-0025] Silvie, O. , Goetz, K. , & Matuschewski, K. (2008). A sporozoite asparagine‐rich protein controls initiation of Plasmodium liver stage development. PLoS Pathogens, 4, e1000086. 18551171 10.1371/journal.ppat.1000086 PMC2398788

[cmi12735-bib-0026] Sinha, A. , Hughes, K. R. , Modrzynska, K. K. , Otto, T. D. , Pfander, C. , Dickens, N. J. , … Waters, A. P. (2014). A cascade of DNA‐binding proteins for sexual commitment and development in Plasmodium. Nature, 507, 253–257. 24572359 10.1038/nature12970 PMC4105895

[cmi12735-bib-0027] Tachibana, S. , Sullivan, S. A. , Kawai, S. , Nakamura, S. , Kim, H. R. , Goto, N. , … Tanabe, K. (2012). Plasmodium cynomolgi genome sequences provide insight into Plasmodium vivax and the monkey malaria clade. Nature Genetics, 44, 1051–1055. 22863735 10.1038/ng.2375 PMC3759362

[cmi12735-bib-0028] Tarun, A. S. , Peng, X. , Dumpit, R. F. , Ogata, Y. , Silva‐Rivera, H. , Camargo, N. , … Kappe, S. H. (2008). A combined transcriptome and proteome survey of malaria parasite liver stages. Proceedings of the National Academy of Sciences of the United States of America, 105, 305–310. 18172196 10.1073/pnas.0710780104 PMC2224207

[cmi12735-bib-0029] Wang, L. , Wang, S. , & Li, W. (2012). RSeQC: Quality control of RNA‐seq experiments. Bioinformatics, 28, 2184–2185. 22743226 10.1093/bioinformatics/bts356

[cmi12735-bib-0030] Waters, A. P. , Higgins, D. G. , & McCutchan, T. F. (1993). Evolutionary relatedness of some primate models of Plasmodium. Molecular Biology and Evolution, 10, 914–923. 7689135 10.1093/oxfordjournals.molbev.a040038

[cmi12735-bib-0031] Westenberger, S. J. , McClean, C. M. , Chattopadhyay, R. , Dharia, N. V. , Carlton, J. M. , Barnwell, J. W. , … Winzeler, E. A. (2010). A systems‐based analysis of plasmodium vivax lifecycle transcription from human to mosquito. PLoS Neglected Tropical Diseases, 4, e653. 20386602 10.1371/journal.pntd.0000653 PMC2850316

[cmi12735-bib-0032] Young, M. D. , Wakefield, M. J. , Smyth, G. K. , & Oshlack, A. (2010). Gene ontology analysis for RNA‐seq: Accounting for selection bias. Genome Biology, 11, R14. 20132535 10.1186/gb-2010-11-2-r14 PMC2872874

[cmi12735-bib-0033] Yuda, M. , Iwanaga, S. , Kaneko, I. , & Kato, T. (2015). Global transcriptional repression: An initial and essential step for Plasmodium sexual development. Proceedings of the National Academy of Sciences of the United States of America, 112, 12824–12829. 26417110 10.1073/pnas.1504389112 PMC4611670

[cmi12735-bib-0034] Zhang, M. , Fennell, C. , Ranford‐Cartwright, L. , Sakthivel, R. , Gueirard, P. , Meister, S. , … Nussenzweig, V. (2010). The Plasmodium eukaryotic initiation factor‐2alpha kinase IK2 controls the latency of sporozoites in the mosquito salivary glands. The Journal of Experimental Medicine, 207, 1465–1474. 20584882 10.1084/jem.20091975 PMC2901070

[cmi12735-bib-0035] Zhu, L. , Mok, S. , Imwong, M. , Jaidee, A. , Russell, B. , Nosten, F. , … Bozdech, Z. (2016). New insights into the plasmodium vivax transcriptome using RNA‐seq. Scientific Reports, 6, 20498. 26858037 10.1038/srep20498 PMC4746618

