# Supplementary material for: Laser capture microdissection enables transcriptomic analysis of dividing and quiescent liver stages of Plasmodium relapsing species
Source: Cell Microbiol. 2017 Mar 13;19(8):e12735. doi: 10.1111/cmi.12735 (PMC5516136; doi:10.1111/cmi.12735)
Supplement: Supplementary file 1 — Data S1. Supporting info item [file CMI-19-na-s001.docx]

**Supplementary Information for Cubi *et al*, 2017**

Contains Supplementary Materials and Methods, Supplementary Figures S1-S6

**Supplementary Materials and Methods**

***Ethics statement***

Adult cynomolgus macaques (*Macaca fascicularis*) were imported from Mauritius and housed in the Infectious Disease Models for Innovative Therapies (IDMIT) facilities at the Fontenay-aux-Roses research center of the Commissariat d’Energie Atomique (CEA). They were used at the IDMIT in accordance with the French national regulation, under the supervision of national veterinary inspectors. IDMIT facilities are in compliance with the standards for human care and use of laboratory animals (Animal welfare Assurance, OLAW number #A5826-01). The use of non-human primates at CEA is in accordance with recommendations of the Weatherall report. Experimental procedures were conducted in strict accordance with the recommendations of the European guidelines for the care and use of laboratory animals (European directive 63/210). The protocols and the use of hepatocytes for the purpose of the work described herein were approved by the Ethical Animal Committee of the CEA.

***Preparation of P. cynomolgi sporozoite stages***

*M. fascicularis* adults were infected with the *P. cynomolgi* M strain by injection of frozen infected blood of *M. fascicularis* or by intra-peritoneal injection of 1×10^6^ sporozoites. Female *A. stephensi* mosquitoes were fed a blood meal of an infected splenectomized *M. fascicularis*, using the Hemotek 5W1 membrane feeding system (Hemotek Ltd). 14–35 days after the blood meal, *P. cynomolgi* sporozoites were extracted from mosquito salivary glands, which were removed by hand dissection and crushed in a potter. The parasites were recovered after filtration through a 40-μm filter (Cell Strainer, Becton Dickinson) and a 2 min centrifugation at 15000g at 4°C.

***Culture of M. fascicularis primary hepatocytes in vitro***

Simian hepato­cytes were isolated from liver segments collected from healthy *M. fascicularis* involved in unrelated studies that required euthanasia. All hepatocytes were isolated using collagenase perfusion as previously described (Dembele et al., 2011). Briefly, the hepatic segments were successively perfused at room temperature at a constant flow rate of around 1 ml per gram of tissue per min, according to the size of the hepatic fragment, with 500 ml of 50 mM HEPES and 0.5 mM EGTA in HBSS buffer (Life Technologies SAS, Saint-Aubain, France), then with 250 ml HBSS-HEPES, and finally with 200 ml 0.02% collagenase type IV (Sigma-Aldrich, Saint-Quentin Fallavier, France) and 0.05% CaCl_2_ in HBSS-HEPES buffer, with recirculation, until appearance of marbling and tissue softening. All buffers were pre-warmed to 37°C. The hepatic fragments were then dissociated in a vessel and viable cells recovered after filtration through a 40 µm-mesh Cell Strainer (BD Biosciences) and cen­trifugation over a 40% Percoll cushion for 3 min at room temperature at 750g. The resulting simian primary hepatocytes (in the pellet) were immediately cryopreserved with a Nicool-FREEZAL controlled-rate freezer (Air Liquide Santé, Marnes La Vallée, France) at a rate of −2 °C per min until −4°C, then at −1°C per min until −40°C with an automatic controlled seeding point at −4°C and finally at −10°C per min until −140°C. Once at −140°C, the cryovials were transferred for storage in liquid nitrogen. When needed for infection, the cryopreserved hepatocytes were subjected to fast thawing at 37°C and cultured in William’s Medium E (Life Technologies) supplemented with 10% FCS (Perbio), 50 µM hydrocortisone hemisuccinate (Upjohn Laboratories SERB, France), 5 µg/ml insulin (Sigma), 2 mM l-glutamine, 200 U/ml penicillin, 200 µg/ml streptomycin (Life Technologies) and Matrigel at 140 mg/ml (BD Biosciences). Cultures were incubated at 37°C with 5% CO_2_.

***Laser capture microdissection (LCM)***

*P. cynomolgi*-infected hepatocytes were microdissected at day 7 post-infection. The schizont forms were visualized using Cresyl violet staining (Sigma). Briefly, the hepatocyte cultures were dehydrated in an ethanol concentration series (95% for 15 s, 75% for 15 s and 70% for 15 s), following which they were incubated for 45 s in 1% Cresyl violet prepared in 70% ethanol w/v. A final round of dehydration was performed with a reverse ethanol concentration series of 70%, 75%, 95% and 2x 100%, with an incubation of 15 s in each solution (Fig. S1B). LCM was carried out using the PALM microlaser system (Zeiss) and the PALM-Robo software, which permits the selecton, posterior cut and catapulting of regions of interest into a collection tube. 1 to 100 schizont stage parasites were captured and pooled for RNA preparation as described in the main text. Following schizont capture, a big area of the same culture was microdissected to assess RNA quality.

*P. cynomolgi* hypnozoites were visualized using a rapid immunostaining method optimized for RNA preservation (Fig. S1B). Infected hepatocytes were fixed for 5 min in ice-cold ethanol at -20°C, following which they were stained with a 1:25 dilution of anti–*P. falciparum* HSP70 2E6 monoclonal antibodies, which cross-react with *P. cynomolgi* HSP70 (ref), for 3 minutes at room temperature in RNase-free phosphate buffered saline (PBS) (Gibco) supplemented with 10% bovine serum albumin (BSA; Sigma). The cells were washed in RNase-free PBS and incubated with a 1:25 dilution of Alexa 488–conjugated goat anti-mouse immunoglobulin (Invitrogen) for 3 minutes at room temperature in PBS with 10% BSA. Immunostained parasites were visualized using indirect fluorescence in the PALM microlaser system and 30-50 hypnozoite stage parasites captured and pooled for RNA extraction as described in the main text. Following hypnozoite capture, a big area of the same culture was microdissected to assess RNA quality. Note that all solutions used for hypnozoite immunostaining were supplemented with 40 U/ml RNase inhibitor (Roche).

***Bioinformatic analysis***

Blastp (Altschul et al., 1990) was used to identify protein sequences in *P. falciparum, P. vivax, P. berghei* and *P. cynomolgi* that are closely related to the PCYB_021650-encoded serine/threonine kinase. The 50 top hits were aligned using Clustal omega (McWilliam et al., 2013), the clustal file analyzed in the http://phylogeny.fr website (Dereeper et al., 2008) using the modules PhyML for tree construction (Anisimova and Gascuel, 2006; Guindon et al., 2010) and Treedyn for tree visualization (Chevenet et al., 2006).

**Supplementary Figures S1-S6**

**
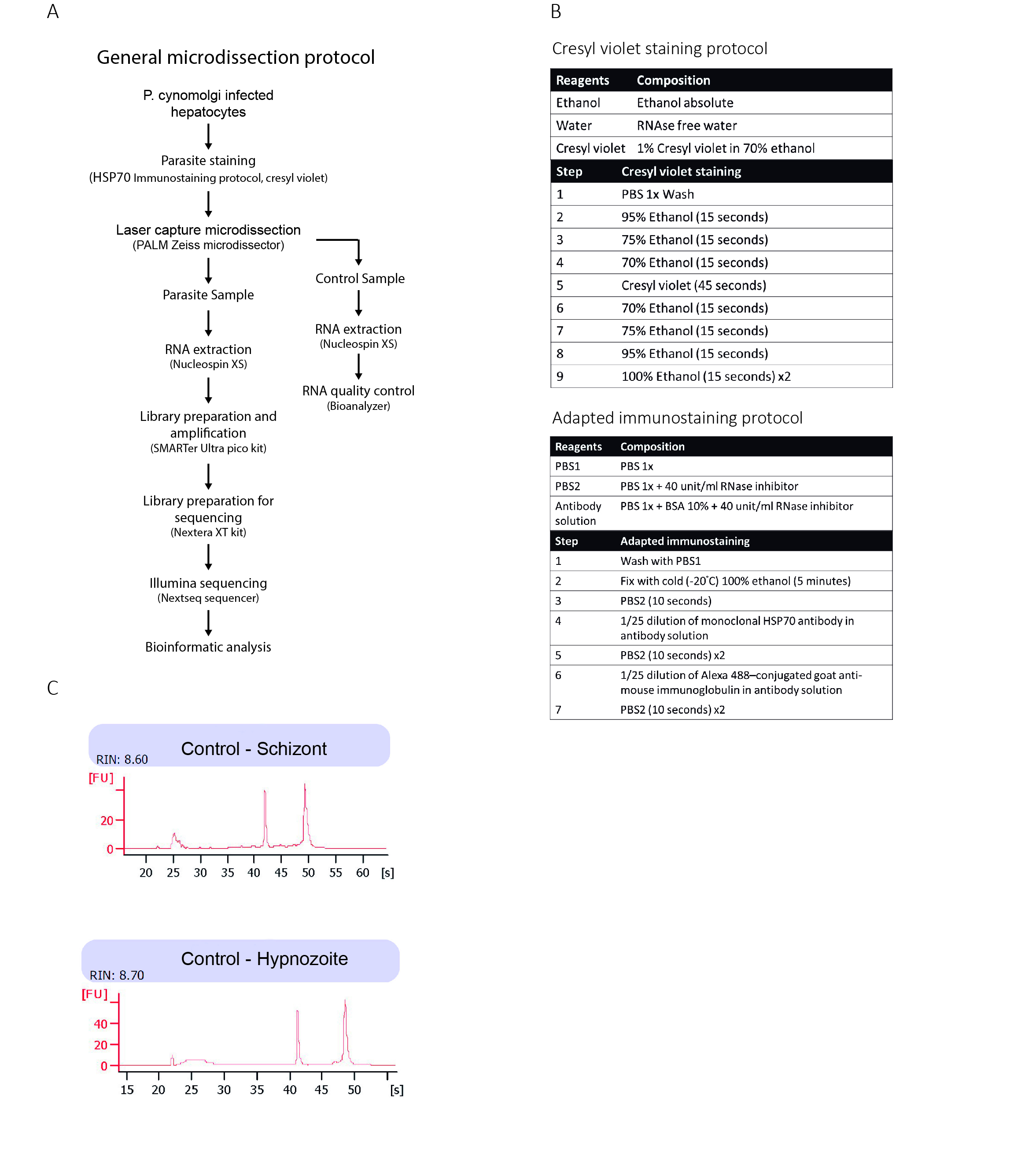
Figure S1: A.** Schematic representation of the different steps of the Laser Capture Microdissection (LCM) and RNA-seq library preparation protocol for *Plasmodium* *cynomolgi* liver stages. **B.** Optimized LCM-adapted Cresyl violet staining protocol for the detection of liver schizonts (upper panel) and immunostaining protocol for the detection of hypnozoites (lower panel). **C.** Representative electropherogram of RNA extracted from a control microdissected area for the schizont (upper panel) and hypnozoite (lower panel) samples showing the RNA Integrity Number (RIN). Samples with a RIN>8 were chosen for RNA-seq library preparation.


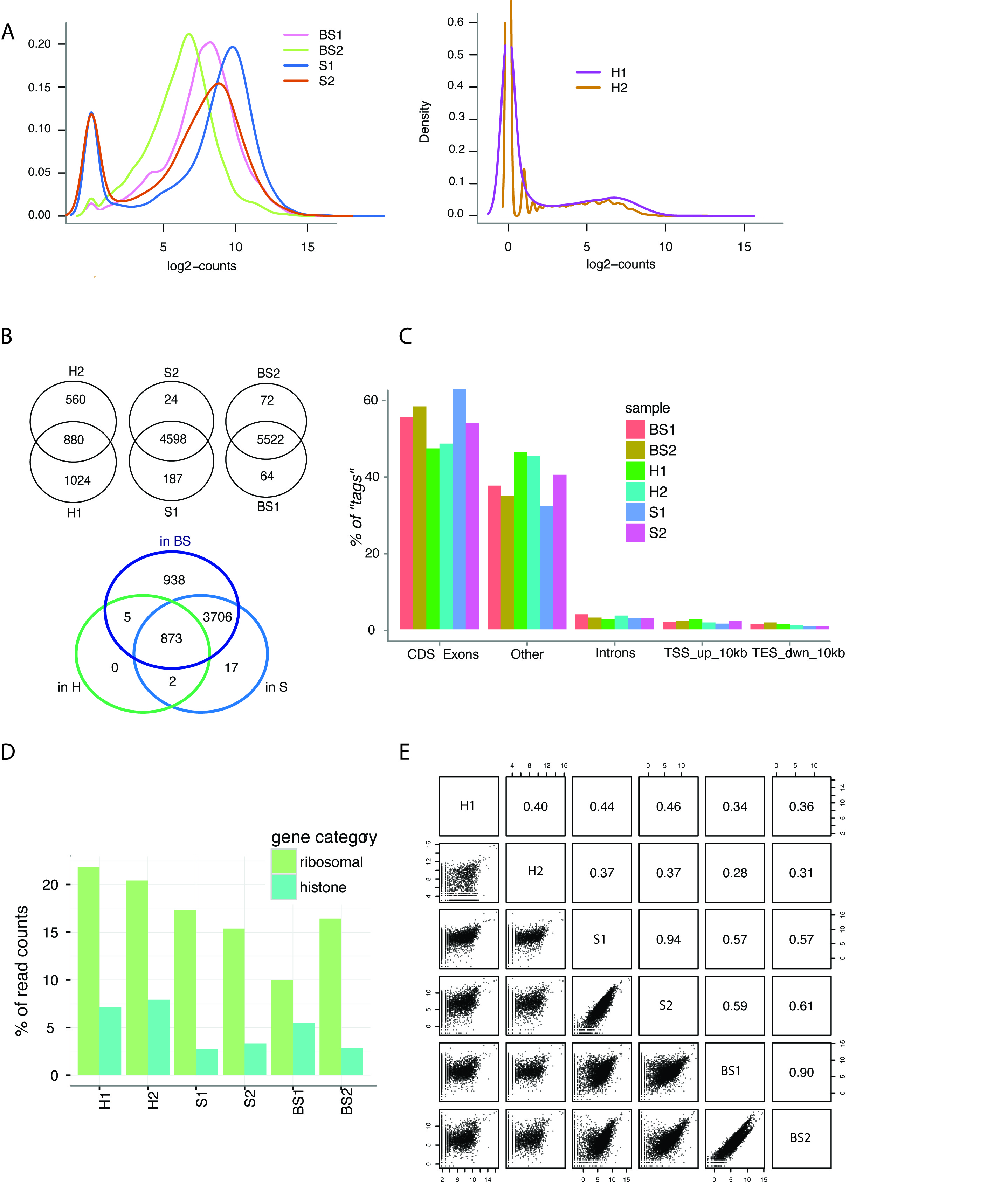


**Figure S2: A.** Density distribution of the read counts for the four samples from dividing stages (Schizont “S” and Blood stage “BS”, left panel) and the two biological replicates of the non-dividing hypnozoite stage (“H”, right panel). **B.** Venn diagram showing the number of genes with at least 1 read count per million (CPM) in the two biological replicates of each stage and the overlap in genes with at least 1 CPM in all samples. **C.** Distribution of reads across annotated regions of the *P. cynomolgi* genome. **D.** Percentage of reads mapping to *P. cynomolgi* ribosomal or histone genes. **E.** Scatter plots showing all pairwise log_2_CPM correlations between the six samples. The upper right part of the panel shows the value of the calculated Pearson correlation coefficients.

**
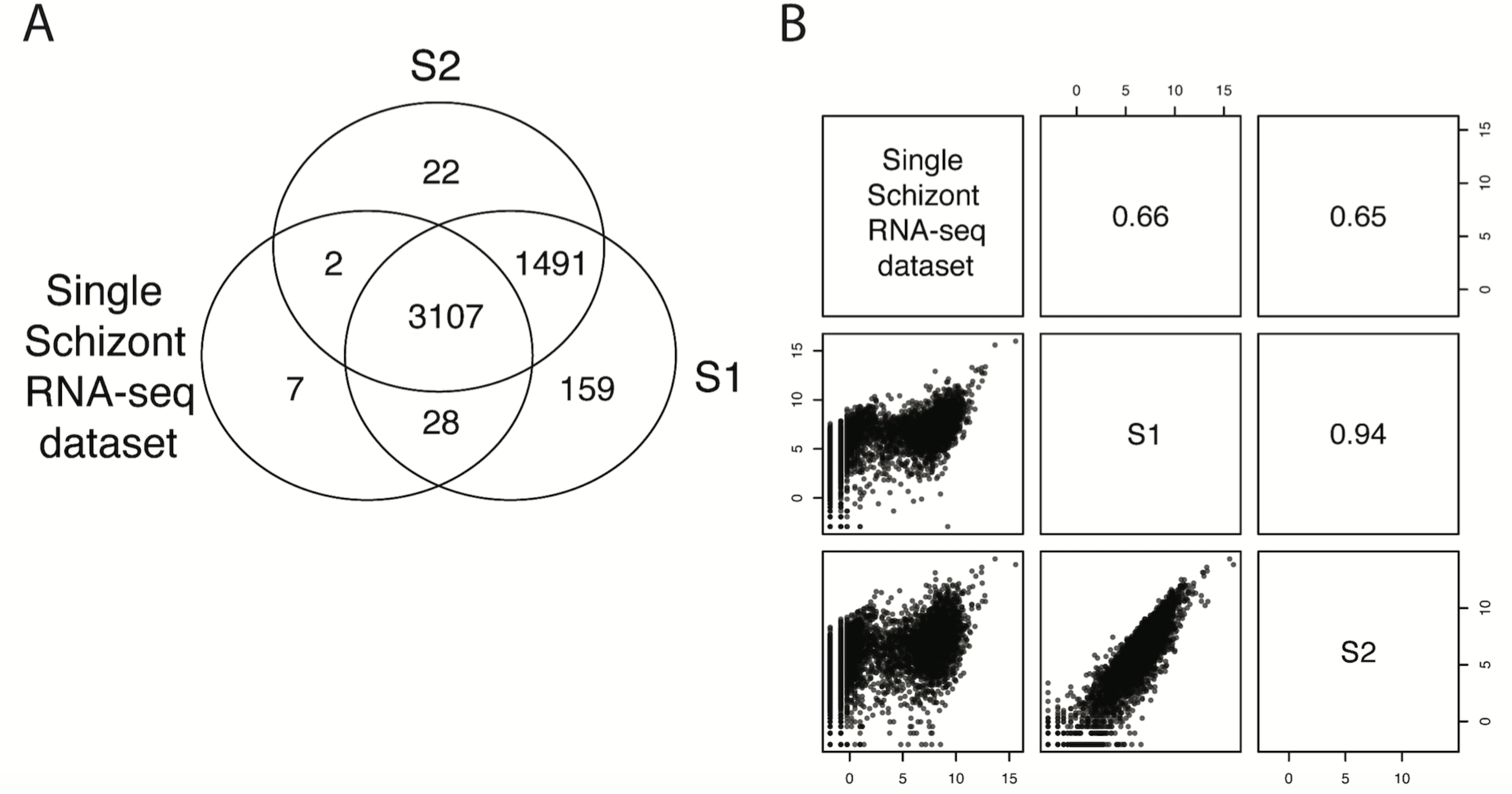
**

**Figure S3:** **A.** Venn diagram showing the number of genes with at least 1 CPM in the two biological replicates of the *P. cynomolgi* liver schizont (pooled samples S1 and S2) and the single liver schizont. **B.** Scatter plots showing all pairwise log_2_CPM correlations between S1, S2 and the single schizont sample. The upper right part of the panel shows the value of the calculated Pearson correlation coefficients.

**
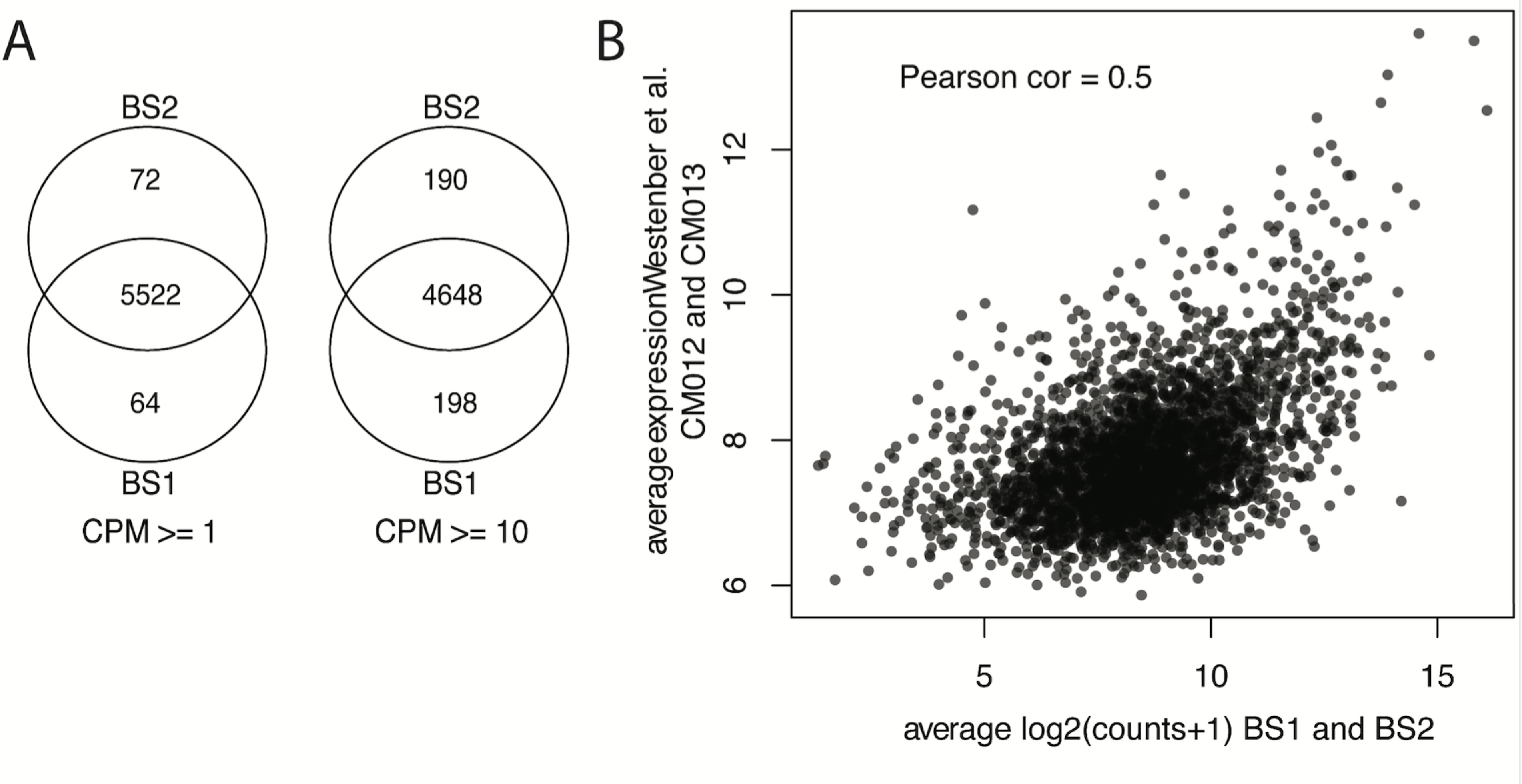
**

**Figure S4: A.** Venn diagram showing the number of genes with at least 1 CPM (left) or 10 CPM (right) in the two biological replicates of the *P. cynomolgi* blood stage samples. **B.** Comparison of the gene expression between *P. cynomolgi* blood stages (average log_2_(read counts + 1) of the two replicates; x-axis) and the microarray data from Westenberger *et al* (ref) for *P. vivax* blood stages (average of samples CM012 and CM013; y=axis) .

**
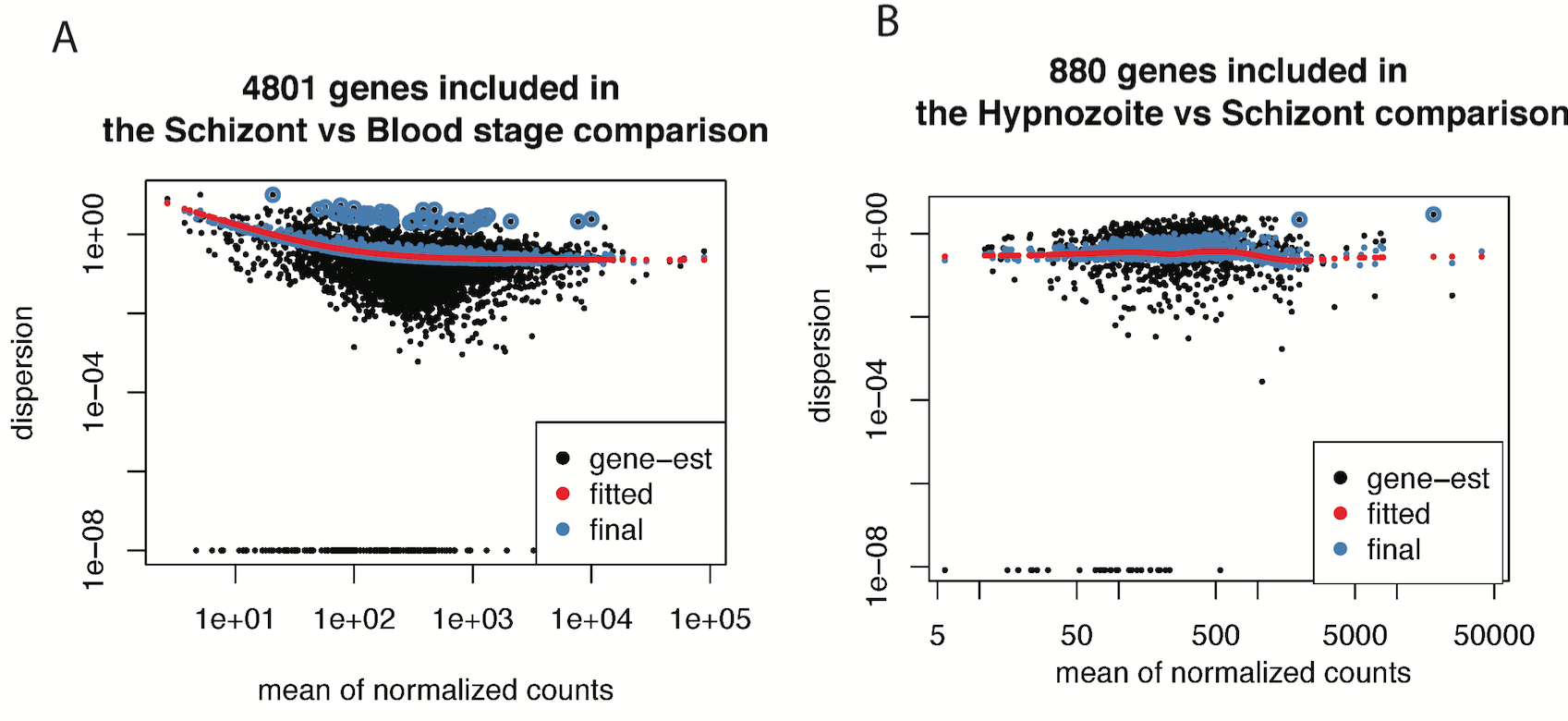
**

**Figure S5:** Plots of the DESeq2 per-gene dispersion estimates together with the fitted mean-dispersion relationship for: **A.** The 4801 genes selected for the Schizont vs Blood Stage comparison, and B. The 880 genes selected for the Hypnozoite and Schizont comparison.


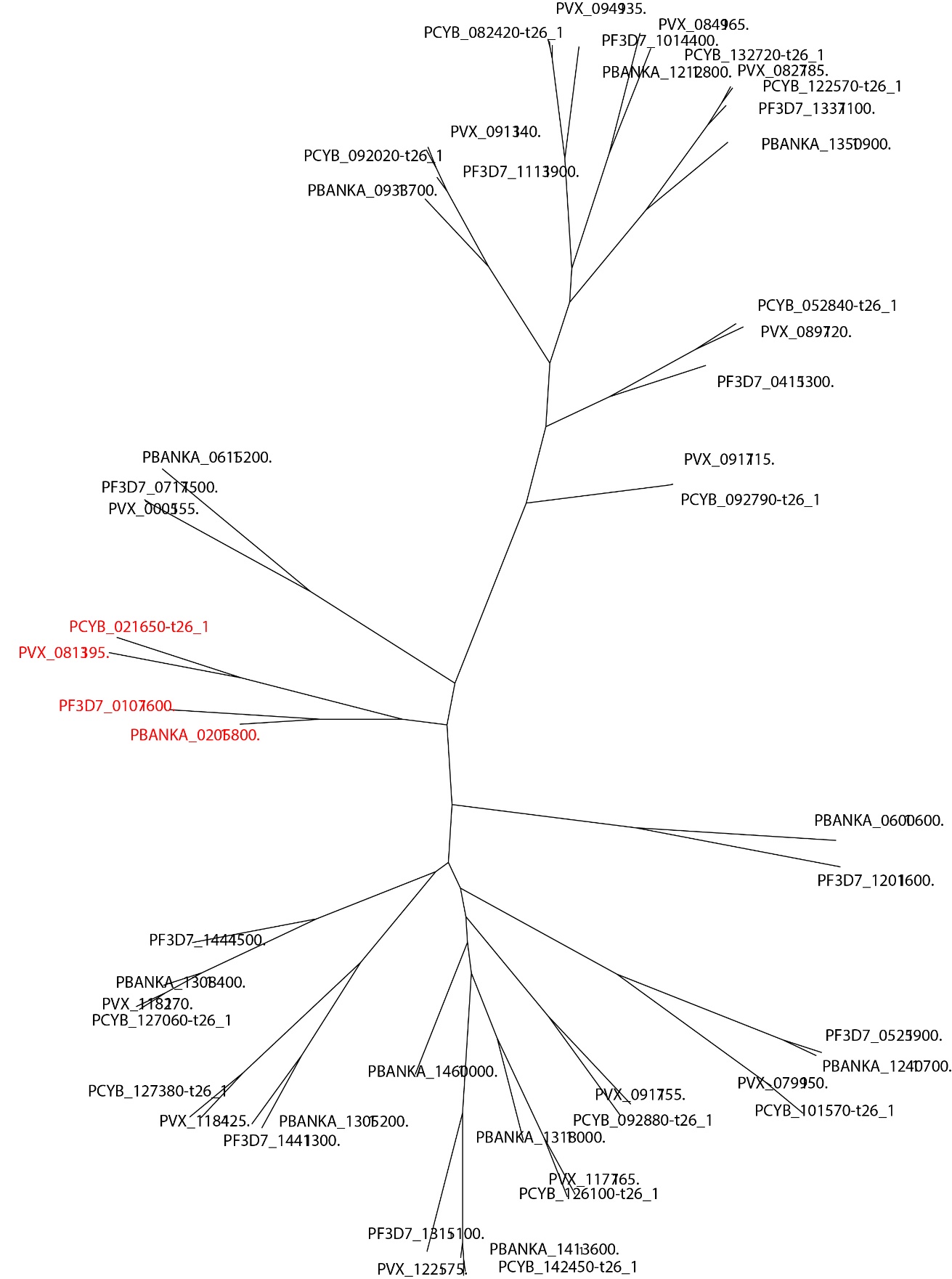


**Figure S6:** Phylogenetic tree of the top 50 *P. falciparum, P. vivax, P. berghei* and *P. cynomolgi* hits obtained from Blastp analysis of serine/threonine kinase encoded by the *PCYB_021650*. Highlighted in red is the eIF2α kinase eIK2 group containing *PCYB_021650* and its putative *P. falciparum*, *P. vivax* and *P. berghei* orthologs. The tree was constructed using the Treedyn phylogeny representation software.

**LIST OF REFERENCES**

Altschul, S.F., Gish, W., Miller, W., Myers, E.W., and Lipman, D.J. (1990) Basic local alignment search tool. *J Mol Biol* **215**: 403-410.

Anisimova, M., and Gascuel, O. (2006) Approximate likelihood-ratio test for branches: A fast, accurate, and powerful alternative. *Syst Biol* **55**: 539-552.

Chevenet, F., Brun, C., Bañuls, A.L., Jacq, B., and Christen, R. (2006) TreeDyn: towards dynamic graphics and annotations for analyses of trees. *BMC Bioinformatics* **7**: 439.

Dembele, L., Gego, A., Zeeman, A.M., Franetich, J.F., Silvie, O., Rametti, A., Le Grand, R., Dereuddre-Bosquet, N., Sauerwein, R., van Gemert, G.J., Vaillant, J.C., Thomas, A.W., Snounou, G., Kocken, C.H., and Mazier, D. (2011) Towards an in vitro model of Plasmodium hypnozoites suitable for drug discovery. *PLoS One* **6**: e18162.

Dereeper, A., Guignon, V., Blanc, G., Audic, S., Buffet, S., Chevenet, F., Dufayard, J.F., Guindon, S., Lefort, V., Lescot, M., Claverie, J.M., and Gascuel, O. (2008) Phylogeny.fr: robust phylogenetic analysis for the non-specialist. *Nucleic Acids Res* **36**: W465-9.

Guindon, S., Dufayard, J.F., Lefort, V., Anisimova, M., Hordijk, W., and Gascuel, O. (2010) New algorithms and methods to estimate maximum-likelihood phylogenies: assessing the performance of PhyML 3.0. *Syst Biol* **59**: 307-321.

McWilliam, H., Li, W., Uludag, M., Squizzato, S., Park, Y.M., Buso, N., Cowley, A.P., and Lopez, R. (2013) Analysis Tool Web Services from the EMBL-EBI. *Nucleic Acids Res* **41**: W597-600.
