# Supplementary material for: Laser capture microdissection enables transcriptomic analysis of dividing and quiescent liver stages of Plasmodium relapsing species
Source: Cell Microbiol. 2017 Mar 13;19(8):e12735. doi: 10.1111/cmi.12735 (PMC5516136; doi:10.1111/cmi.12735)

A

General microdissection protocol

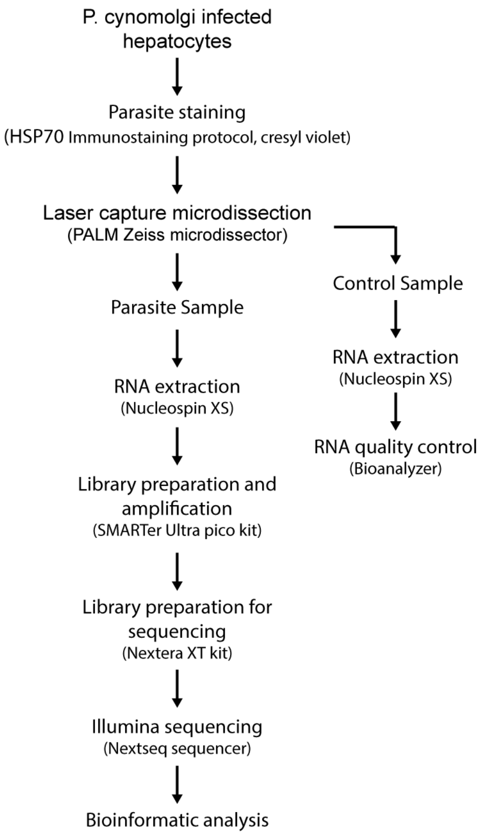

B

Cresyl violet staining protocol

Reagents	Composition
Ethanol	Ethanol absolute
Water	RNAse free water
Cresyl violet	1% Cresyl violet in 70% ethanol
**Step**	**Cresyl violet staining**
1	PBS 1x Wash
2	95% Ethanol (15 seconds)
3	75% Ethanol (15 seconds)
4	70% Ethanol (15 seconds)
5	Cresyl violet (45 seconds)
6	70% Ethanol (15 seconds)
7	75% Ethanol (15 seconds)
8	95% Ethanol (15 seconds)
9	100% Ethanol (15 seconds) x2

Adapted immunostaining protocol

Reagents	Composition
PBS1	PBS 1x
PBS2	PBS 1x + 40 unit/ml RNase inhibitor
Antibody solution	PBS 1x + BSA 10% + 40 unit/ml RNase inhibitor
**Step**	**Adapted immunostaining**
1	Wash with PBS1
2	Fix with cold (-20°C) 100% ethanol (5 minutes)
3	PBS2 (10 seconds)
4	1/25 dilution of monoclonal HSP70 antibody in antibody solution
5	PBS2 (10 seconds) x2
6	1/25 dilution of Alexa 488–conjugated goat anti-mouse immunoglobulin in antibody solution
7	PBS2 (10 seconds) x2

C

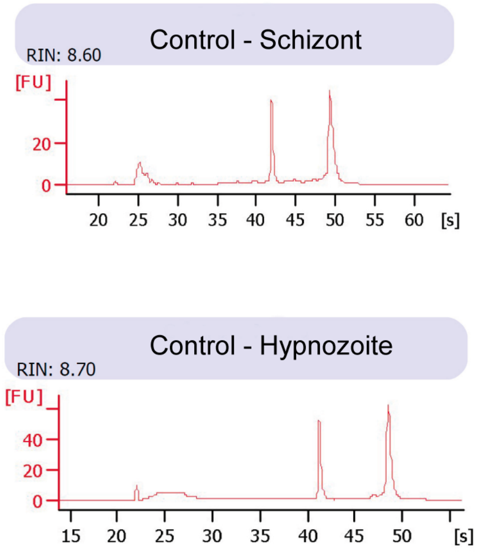

Supplement: Supplementary file 2 — Figure S1: A. Schematic representation of the different steps of the Laser Capture Microdissection (LCM) and RNA‐seq library preparation protocol for Plasmodium cynomolgi liver stages. B. Optimized LCM‐adapted Cresyl violet staining protocol for the detection of liver schizonts (upper panel) and immunostaining protocol for the detection of hypnozoites (lower panel). C. Representative electropherogram of RNA extracted from a control microdissected area for the schizont (upper panel) and hypnozoite (lower panel) samples showing the RNA Integrity Number (RIN). Samples with a RIN > 8 were chosen for RNA‐seq library preparation. [file CMI-19-na-s002.pdf]
