# Supplementary figures and images for: Laser capture microdissection enables transcriptomic analysis of dividing and quiescent liver stages of Plasmodium relapsing species
Source: Cell Microbiol. 2017 Mar 13;19(8):e12735. doi: 10.1111/cmi.12735 (PMC5516136; doi:10.1111/cmi.12735)

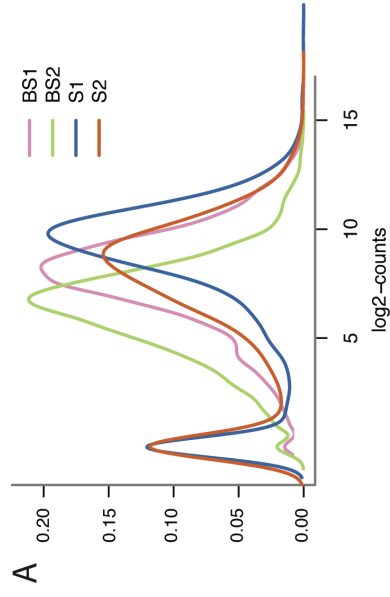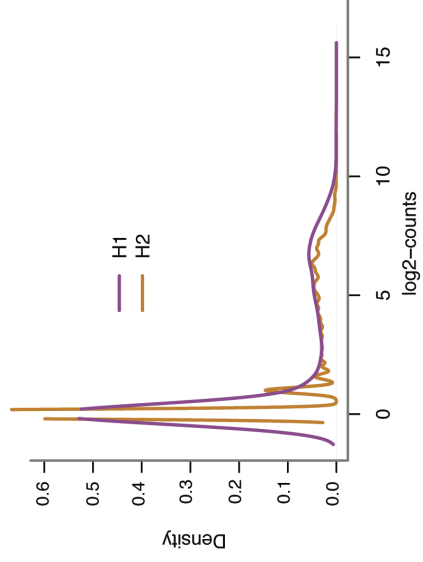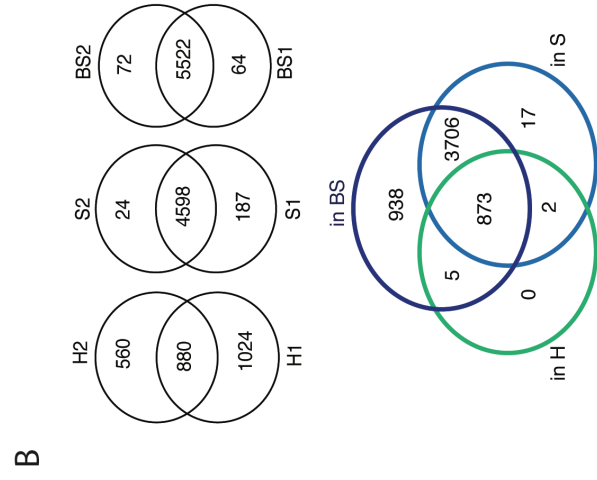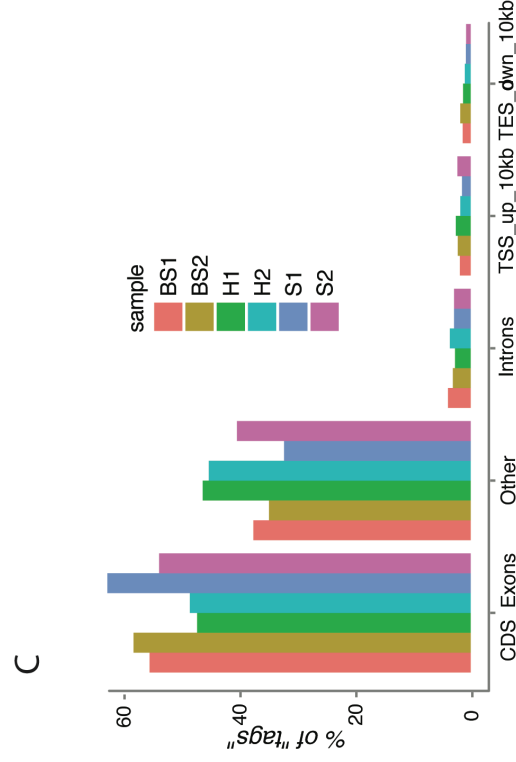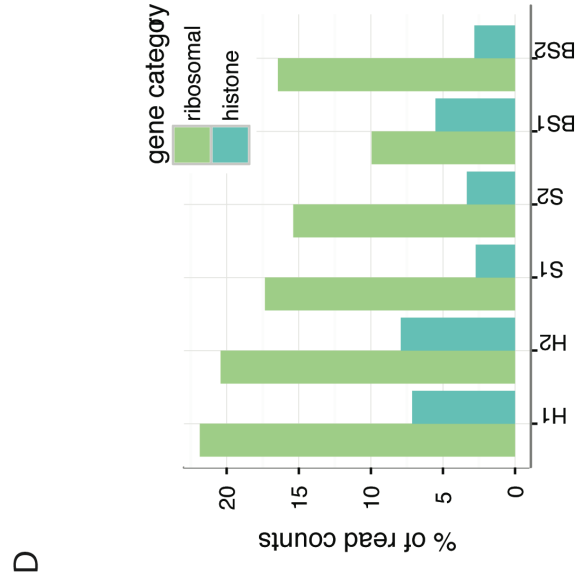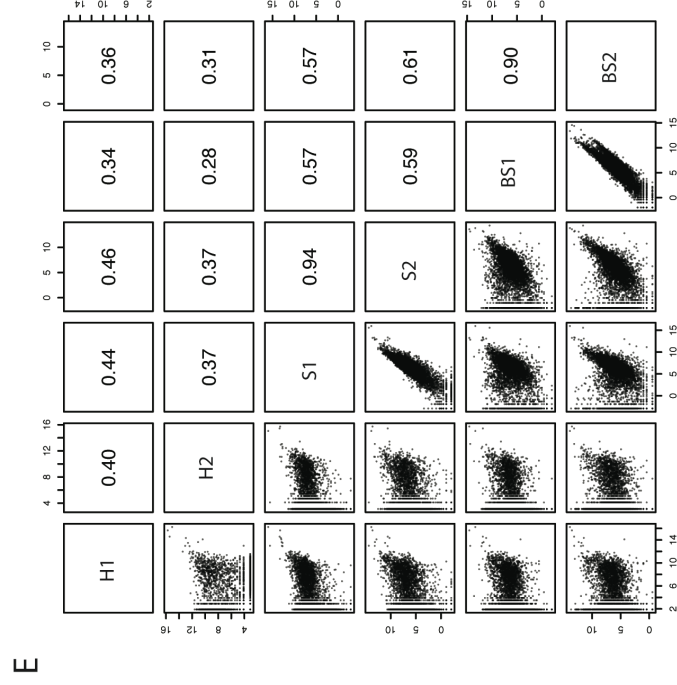

Supplement: Supplementary file 3 — Figure S2: A. Density distribution of the read counts for the four samples from dividing stages (Schizont “S” and Blood stage “BS”, left panel) and the two biological replicates of the non‐dividing hypnozoite stage (“H”, right panel). B. Venn diagram showing the number of genes with at least 1 read count per million (CPM) in the two biological replicates of each stage and the overlap in genes with at least 1 CPM in all samples. C. Distribution of reads across annotated regions of the P. cynomolgi genome. D. Percentage of reads mapping to P. cynomolgi ribosomal or histone genes. E. Scatter plots showing all pairwise log2CPM correlations between the six samples. The upper right part of the panel shows the value of the calculated Pearson correlation coefficients. [file CMI-19-na-s003.pdf]

A

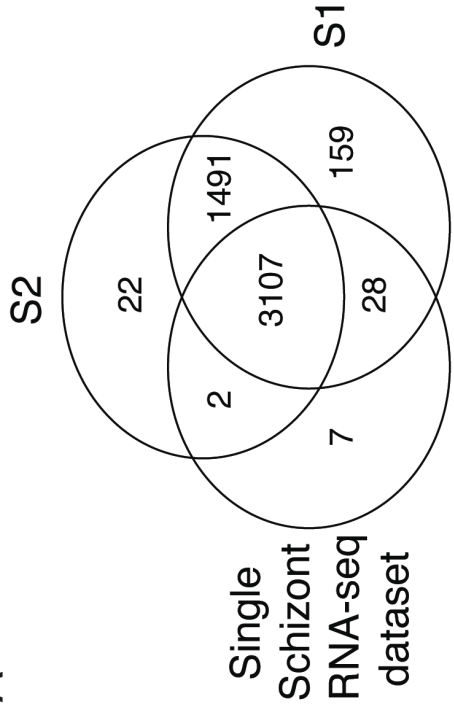

B

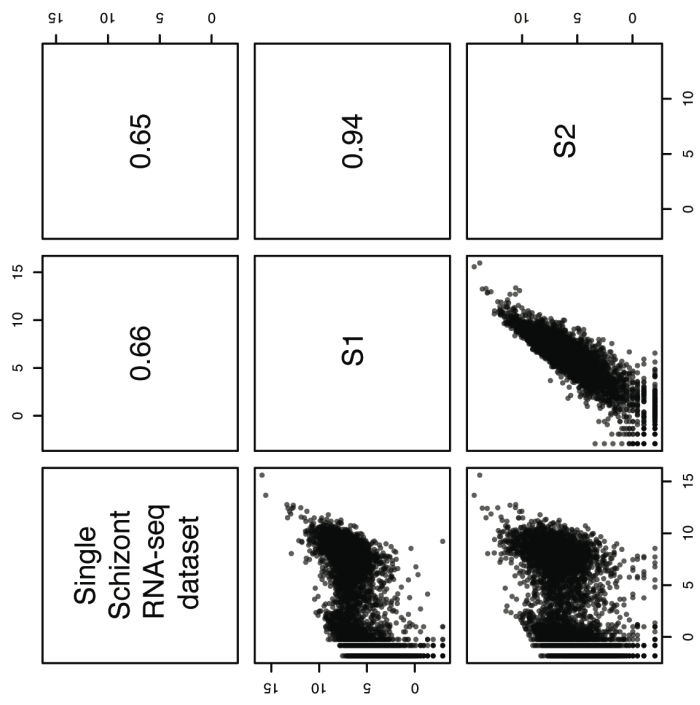

Supplement: Supplementary file 4 — Figure S3: A. Venn diagram showing the number of genes with at least 1 CPM in the two biological replicates of the P. cynomolgi liver schizont (pooled samples S1 and S2) and the single liver schizont. B. Scatter plots showing all pairwise log2CPM correlations between S1, S2 and the single schizont sample. The upper right part of the panel shows the value of the calculated Pearson correlation coefficients. [file CMI-19-na-s004.pdf]

A

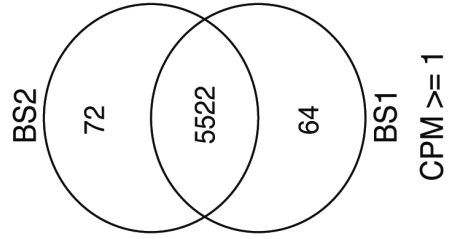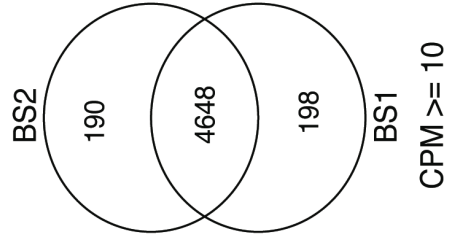

B

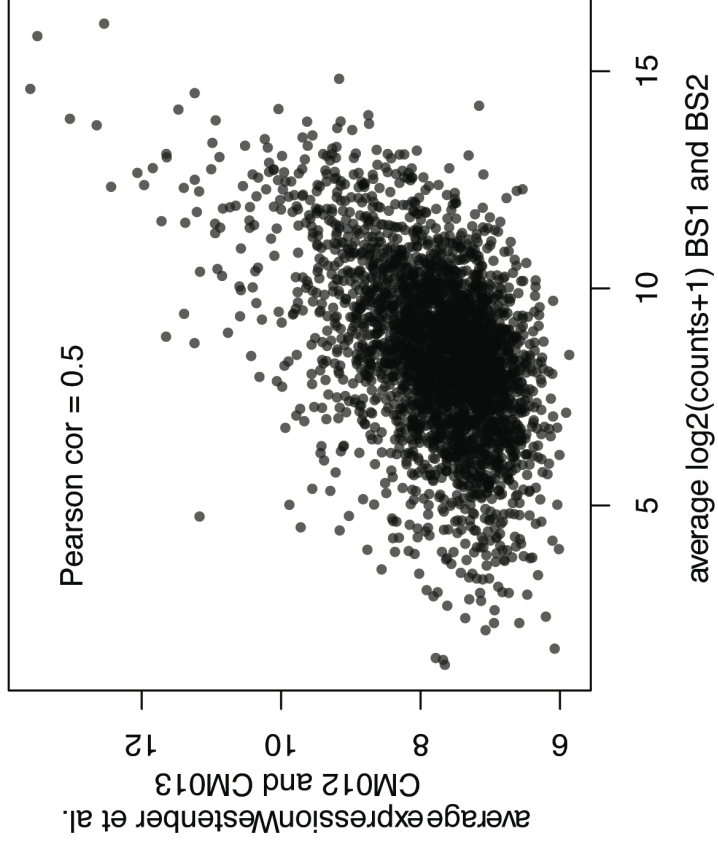

Supplement: Supplementary file 5 — Figure S4: A. Venn diagram showing the number of genes with at least 1 CPM (left) or 10 CPM (right) in the two biological replicates of the P. cynomolgi blood stage samples. B. Comparison of the gene expression between P. cynomolgi blood stages (average log2(read counts +1) of the two replicates; x‐axis) and the microarray data from Westenberger et al. (ref) for P. vivax blood stages (average of samples CM012 and CM013; y = axis). [file CMI-19-na-s005.pdf]

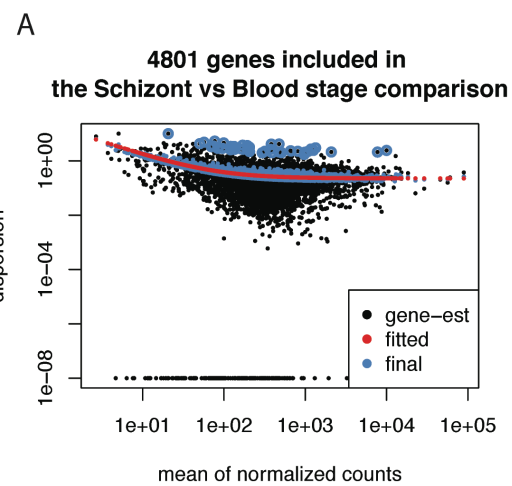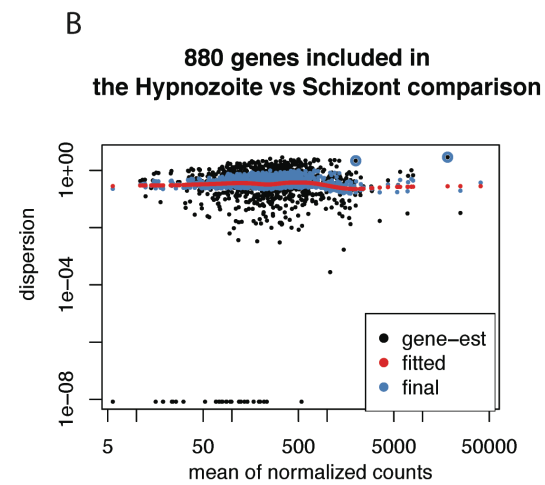

Supplement: Supplementary file 6 — Figure S5: Plots of the DESeq2 per‐gene dispersion estimates together with the fitted mean‐dispersion relationship for: A. The 4801 genes selected for the Schizont vs Blood Stage comparison, and B. The 880 genes selected for the Hypnozoite and Schizont comparison. [file CMI-19-na-s006.pdf]

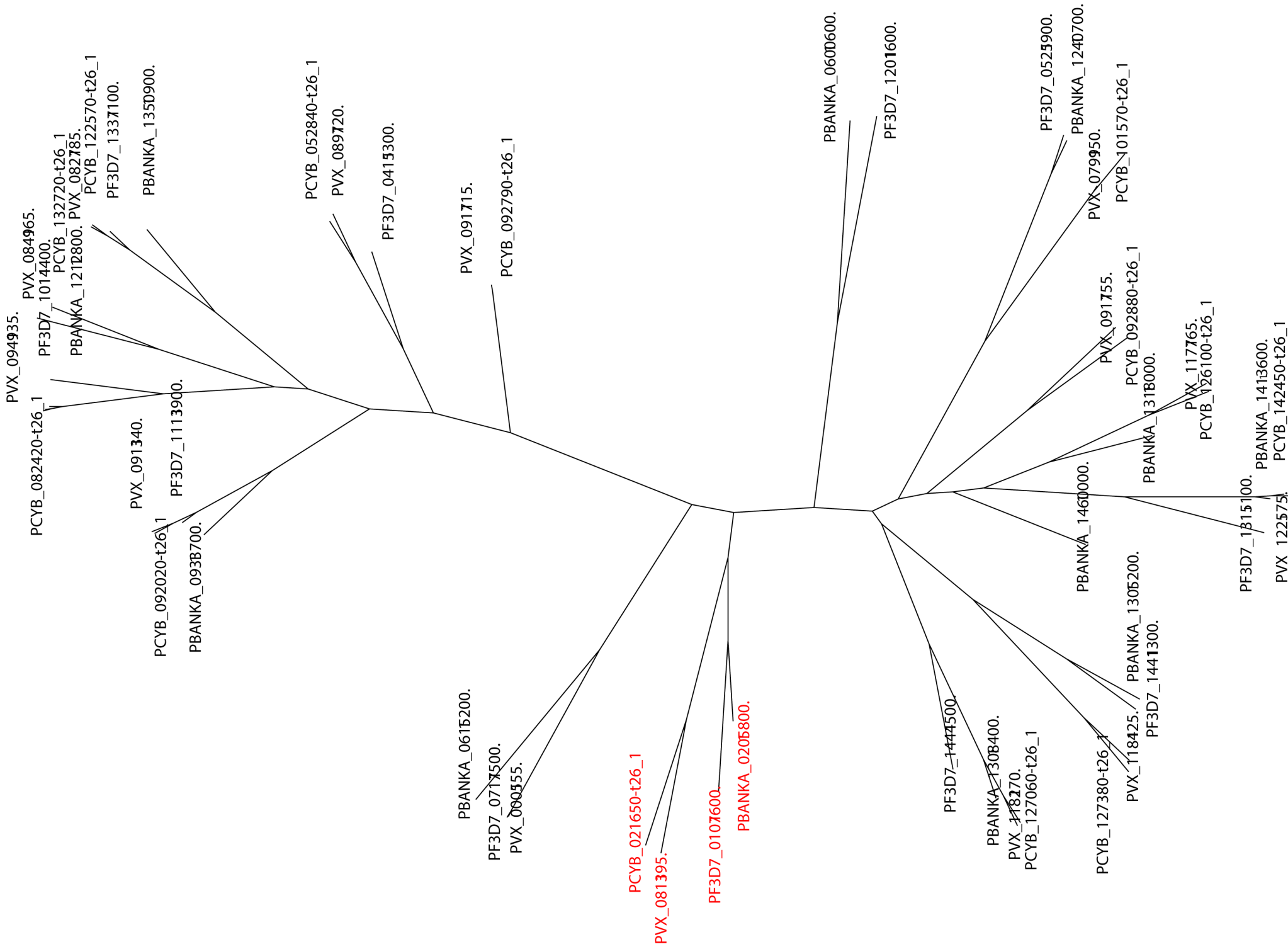

Supplement: Supplementary file 7 — Figure S6: Phylogenetic tree of the top 50 P. falciparum, P. vivax, P. berghei and P. cynomolgi hits obtained from Blastp analysis of serine/threonine kinase encoded by the PCYB_021650. Highlighted in red is the eIF2α kinase eIK2 group containing PCYB_021650 and its putative P. falciparum, P. vivax and P. berghei orthologs. The tree was constructed using the Treedyn phylogeny representation software. [file CMI-19-na-s007.pdf]
